# Dual-stimuli responsive smart nanoprobe for precise diagnosis and synergistic multi-modalities therapy of superficial squamous cell carcinoma

**DOI:** 10.1186/s12951-022-01759-1

**Published:** 2023-01-03

**Authors:** Peisen Zhang, Yingying Cui, Jian Wang, Junwei Cheng, Lichong Zhu, Chuang Liu, Saisai Yue, Runxin Pang, Jiaoqiong Guan, Bixia Xie, Ni Zhang, Meng Qin, Lihong Jing, Yi Hou, Yue Lan

**Affiliations:** 1 Department of Rehabilitation Medicine, Guangzhou First People’s Hospital, School of Medicine, South China University of Technology, 510180 Guangzhou, China; 2 College of Life Science and Technology, Beijing University of Chemical Technology, Beijing, 10029 China; 3 Department of Head and Neck Surgery, National Clinical Research Center for Cancer/Cancer Hospital, National Cancer Center, Chinese Academy of Medical Sciences, Peking Union Medical College, Beijing, 100021 China; 4 Department of Psychiatry, West China Hospital, National Chengdu Center for Safety Evaluation of Drugs, Sichuan University, Chengdu, 610041 China; 5 Key Laboratory of Colloid, Interface and Chemical Thermodynamics, Institute of Chemistry, Chinese Academy of Sciences, Beijing, 100190 China

**Keywords:** Dual-stimuli responsive nanoprobe, Precise diagnosis, Synergistic therapy, Squamous cell carcinoma

## Abstract

**Background:**

Although the promising advancements of current therapeutic approaches is available for the squamous cell carcinoma (SCC) patients, the clinical treatment of SCC still faces many difficulties. The surgical irreparable disfigurement and the postoperative wound infection largely hamper the recovery, and the chemo/radiotherapy leads to toxic side effects.

**Results:**

Herein, a novel pH/Hyaluronidase (HAase) dual-stimuli triggered smart nanoprobe Fe^III^TA@HA has been designed through the biomineralization of Fe^3+^ and polyphenol tannic acid (TA) under the control of hyaluronic acid (HA) matrix. With the HA residues on the outer surface, Fe^III^TA@HA nanoprobes can specifically target the SCC cells through the over-expressed CD44, and accumulate in the carcinoma region after intravenously administration. The abundant HAase in carcinoma microenvironment will trigger the degradation of HA molecules, thereby exposing the Fe^III^TA complex. After ingesting by tumor cells *via* CD44 mediated endocytosis, the acidic lysosomal condition will further trigger the protonation of TA molecules, finally leading to the Fe^3+^ release of nanoprobe, and inducing a hybrid ferroptosis/apoptosis of tumor cells through peroxidase activity and glutathione depletion. In addition, Owing to the outstanding *T*_1_ magnetic resonance imaging (MRI) performance and phototermal conversion efficiency of nanoprobes, the MRI-guided photothermal therapy (PTT) can be also combined to complement the Fe^3+^-induced cancer therapy. Meanwhile, it was also found that the nanoprobes can promote the recruitment of CD4^+^ and CD8^+^ T cells to inhibit the tumor growth through the cytokines secretion. In addition, the Fe^III^TA@HA nanoprobes can be eliminated from the body and no obvious adverse side effect can be found in histological analysis, which confirmed the biosafety of them.

**Conclusion:**

The current Fe^III^TA@HA nanoprobe has huge potential in clinical translation in the field of precise diagnosis and intelligent synergistic therapy of superficial SCC. This strategy will promisingly avoid the surgical defects, and reduce the systemic side effect of traditional chemotherapy, paving a new way for the future SCC treatment.

**Graphical Abstract:**

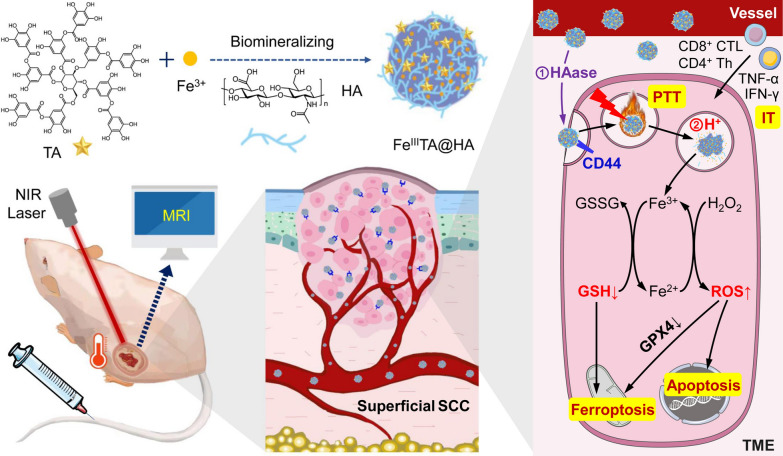

**Supplementary Information:**

The online version contains supplementary material available at 10.1186/s12951-022-01759-1.

## Background

Head and neck cancers are a heterogeneous type of tumors, involving the oral cavity, pharynx, larynx, sinus cavities, orbit, and other related structures like the skin. Owing to a great percentage of these cancers display squamous cell histology, they are referred as squamous cell carcinoma (SCC) [1–4]. Although the current therapeutic approaches of theses cancers take advantage of sophisticated modalities of therapies, the clinical treatment of SCC still faced many difficulties. Owing to the complex anatomy of the head and neck, the complicated multiple procedures of the resection surgery may lead to irreparable disfigurement, and the surgical reconstruction is also a formidable challenge [5–7]. More seriously, the surgical wound is readily contaminated from the oral cavity during the operation, leading to the postoperative wound infection, which is a significant impediment to head and neck SCC recovery [8, 9]. Although the preoperative chemo/radiotherapies have been adopted to combine with surgical operation in order to shrink the tumor and minimize the operative trauma [10], the severe side effects of them cannot be avoided, which even subsequently reduce the postoperative tolerance of patients [11, 12]. Therefore, new treatment strategy of SCC that can not only eradicate the tumor cells but also exhibit a higher safety features is much needed to replace the conventional chemo/radiotherapies.

Apart from the above treatment strategies, because SCC usually occurs in the superficial epidermis or skin appendages, photothermal therapy (PTT) offers a new therapeutic approach for SCC patients, which has higher compliance than the invasive surgery and lower side effects compared with traditional chemo/radiotherapy [13, 14]. In clinical, PTT has been used as a supplement to the present therapeutic approaches of several types of SCC [15]. However, despite the improvements reported before, the appropriate photothermal agent that can not only specifically target but also effectively ablate the tumor cells is still much needed to enhance the efficacy of PTT.

Over the past two decades, a variety of functional nanomaterials have been designed and synthesized to realize the effective cancer diagnosis and treatments [16–20]. Nevertheless, the clinical applications of these nanomedicine still face the biosafety challenges and toxicity concerns because the bio-distributions of the nanomedicine are not confined only to tumors, i.e., a subset of nanomedicine will be inevitably distributed in healthy organs, and indiscriminately attack the healthy cells [21–24].

To address this issue, the concept of stimulus-responsive nanomaterials has been proposed. Such nanomaterials can be activated to exhibit therapeutic efficacy only in the tumor tissues under the stimulation of specific tumor pathological hallmarks, while remaining “silent” in normal tissues to ensure biosafety features [25–29]. For example, in our previous studies, a type of nanoprobes have been developed, which can specifically target the triple-negative breast cancer, be triggered by the acidic microenvironment, and responsively release the Fe^3+^ only in tumor tissues to realize a safer tumor treatment [30]. In another study, Gu and coworkers have developed an ultra-thin and colloidally stable nanosheet with ultra-high 734% doxorubicin loading capacity. The doxorubicin in such nanosheet remains stable under the physiological pH condition, while showing sustained release behavior in the acidic tumor microenvironment and the lysosomes, which possesses superior therapeutic effect of tumor, and minimizes the systemic toxicity as well [31]. Undoubtedly, these smart strategies are promisingly to overcome the toxicity concerns of traditional nanomaterials. Nevertheless, these types of smart theranostic nanomaterials controlled by a single stimulus are still encountered with the limitation of each stimulation, and may not be reliable enough, because the physiological environments of healthy tissues are very complicated, which may lead to unanticipated and accidental activation of nanomaterials. For example, the acidic feature is not specific to the tumor microenvironment. The infectious sites also possess the acidic environment due to the accumulation of lactate [32], which may also trigger the release of the H^+^-responsive nanomaterials and lead to the undesired toxicity [33]. To overcome this limitation, smarter nanomaterials with multiple-stimuli responsive capability should be exploited, which can be triggered only when the two or more stimuli of tumor hallmarks work together. Through the multi-stimuli responsive strategy, the tumor treatment specificity of nanomaterials will be largely enhanced and, more importantly, the unexpected accidental activation of them in normal tissues can be much minimized. Beneficial from these merits, it can be reasonably speculated that the multi-stimuli responsive strategy may be a feasible approach for overcoming the aforementioned clinical challenges of SCC treatment.

**Scheme 1:**
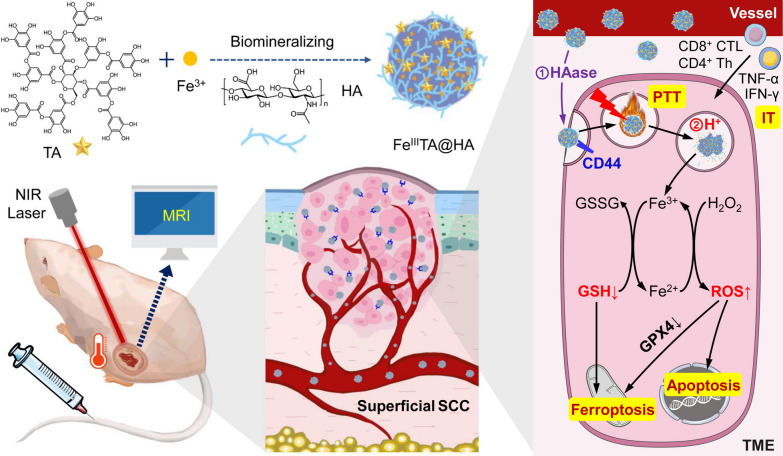
Schematic illustration to demonstrate the synthesis procedure and the theranostics processes of Fe^III^TA@HA nanoprobes through HAase/lysosomal H^+^ dual-stimuli triggered hybrid ferroptosis/apoptosis and MRI-guided precise PTT of superficial squamous cell carcinoma (SCC).

On the basis of recent studies, hyaluronic acid (HA) molecules can not only specifically anchor to CD44 receptor and its different variant isoforms that over-expresses on most types of SCC cells [34–36], but also be degraded into low-molecular-weight fragments by the tumor-associated hyaluronidase (HAase) which abundantly distributes in SCC tumor microenvironment (TME) [37]. Therefore, in the CD44-HA-HAase system, HA can be serve as not only a target molecule for SCC, but also a stimuli-triggered moiety for nanomaterials design. Through making use of this, in this work, a novel pH/HAase dual-stimuli triggered smart nanoprobe Fe^III^TA@HA has been designed and prepared through the biomineralization of Fe^3+^ and polyphenol tannic acid (TA) under the control of HA matrix to realize precise theranostics of SCC. The work details of nanoprobes are given in Scheme 1. With the HA residues on the outer surface, Fe^III^TA@HA nanoprobes are expected to firstly target the CD44 receptors over-expressed on the SCC cells and accumulate in the carcinoma region after intravenously administration. The abundant HAase in carcinoma TME will trigger the degradation of HA molecules, thereby exposing the Fe^III^TA complex. After ingesting by tumor cells *via* CD44 mediated endocytosis, the acidic condition in cell lysosome will further trigger the protonation of TA molecules, leading to the Fe^3+^ release of nanoprobe. These released Fe^3+^ ions are expected to induce ferroptosis/apoptosis of tumor cells through peroxidase activity and glutathione (GSH) depletion. In addition, beneficial from the *T*_1_ MRI performance of Fe^3+^ and outstanding photothermal conversion efficiency of Fe^III^TA complex in nanoprobes, the theranostic strategy of MRI-guided PTT will be also combined to complement the Fe^3+^-induced cancer therapy. Through the synergistic effects of all this approach, the dual-stimuli triggered nanoprobes are expected to effectively diagnose and treat the superficial SCC with satisfactory biosafety features. In the experiments reported below, the construction and characterization of Fe^III^TA@HA nanoprobes are displayed, and the in vitro and in vivo experiments are carried out to verify the dual-stimuli triggered properties, tumor theranostic efficacy, and biosafety of nanoprobes.

## Materials and methods

### Materials

Ferric chloride hexahydrate (FeCl_3_·6H_2_O, 99.0%) was purchased from Shandong Xiya Chemical Industry Company (Shandong, China). Tannic acid (TA, 98%) was purchased from Beijing Innochem Technology Co., Ltd (Beijing, China). Hyaluronic acid (HA, 97%) was purchased from Shanghai Macklin Biochemical Technology Co., Ltd (Shanghai, China). 4-(4,6-dimethoxy-1,3,5-triazin-2-yl)-4-methyl morpholinium chloride (DMTMM) were purchased from Aladdin Co. Ltd. 5-aminofluorescein (5-AF) were purchased from Beijing Innochem Science & Technology co., LTD. All the above chemicals were used without further purification. The human tongue squamous carcinoma SCC-9 cell line was purchased from ATCC (ATCC CRL-1629). CD44 rabbit polyclonal antibody was purchased from Abcam (ab157107).

### Synthesis of Fe^III^TA@HA nanoprobes

Typically, 170 mg TA was dissolved in 25 mL HA solution (32 mg/mL) under vigorous stirring. After 2 h dissolution, 25 mL FeCl_3_ solution (1.08 mg/mL) was added to the reaction mixture and stirred for another 1 h. The resulting aqueous nanoprobes solution was purified with 3 k MWCO centrifugal devices to remove the unreacted materials, then transferred into 1× PBS buffer, and finally stored at 4 °C for further use.

### Characterization of the Fe^III^TA@HA nanoprobes

Transmission electron microscope (TEM) image was carried out with JEM-2100 (UHR) microscopes operating at 200 kV, for characterizing the morphology and size distribution of nanoprobes. The size distribution of nanoprobes was determined by counting more than 100 particles per sample. The UV-Vis absorption spectra were recorded on a microplate reader (Thermo, MULTISKAN GO). DLS measurements were carried out at 298.0 K with Nano ZS (Malvern) equipped with a solid state He-Ne laser (λ = 632.8 nm) for monitoring the hydrodynamic profiles of the particles.

### In vitro photothermal evaluation of Fe^III^TA@HA nanoprobes

To assess the photothermal performance of the Fe^III^TA@HA nanoprobe, a series of aqueous solutions of nanoprobes with different Fe^3+^ concentrations were placed in a 96-well cell culture plate (100 µL/well) to receive 650 nm laser irradiation with different power density for 10 min. The temperature variations were recorded using a digital infrared thermometer per 30 s.

### Relaxivity measurements

The relaxivity measurements were carried out on a 7.0 T animal MRI instrument (Bruker BioSpec70/20 USR). A series of aqueous solutions containing Fe^III^TA@HA nanoprobes were prepared, and 200 µL of each was transferred into Eppendorf tube for MR studies. The *T*_1_ and *T*_2_ relaxation time were recoded through *T*_1_map and *T*_2_map sequences.

The detailed parameters for MR studies were set as follows:

*T*_1_-weighted imaging: echo time (TE) = 5.01 ms, repetition time (TR) = 300 ms, field of view (FoV) = 35 mm × 35 mm, slice thickness = 1 mm.

*T*_1_map: TE = 5.90 ms, TR = 3000, 1500, 1000, 500, 298 ms, FoV = 35 mm × 35 mm, slice thickness = 1 mm.

*T*_2_map: TE = 6.6, 13.2, 19.8, 26.4, 33, 39.6, 46.2, 52.8, 59.4, 66, 72.6, 79.2 ms, TR = 3000 ms, FoV = 35 mm × 35 mm, slice thickness = 1 mm.

### Determination of Fe^3+^ release

The amount of Fe^3+^ released by Fe^III^TA@HA nanoprobes was determined by the Prussian Blue method. Briefly, the nanoprobes (4 mM with respect to Fe) were incubated under different pH conditions in the presence or absence of HAase (76 U/mL), and the released Fe^3+^ ions were collected through ultrafiltration treatment (Millipore YM-3, 3 kD), which then detected through Prussian Blue color reaction, respectively. Fe^3+^ concentration was calculated by measuring the absorption of products at 700 nm.

### Peroxidase-like activity evaluation

The peroxidase-like activity of nanoprobes was evaluated through methylene blue (MB) degradation assay. In detail, 10 µg mL^− 1^ MB was mixed with Fe^III^TA@HA nanoprobes dispersions (0.2 mM with respect to Fe^3+^) in the absence of 1 × 10^− 2^ M H_2_O_2_ under different pH in the presence or absence of HAase (76 U/mL), respectively. The absorbance at 665 nm was measured at different time points.

### Cell culture

SCC-9 cell line was cultured in a medium of DMEM supplemented with 10% fetal bovine serum and 1% penicillin-streptomycin solution (100×) at 37 °C under a 5% CO_2_ atmosphere.

### CD44 blocking efficiency evaluation

HA (80 mg) was dissolved in 5 mL K_2_HPO_4_ aqueous solution (pH 9.1) through magnetic stirring in a round bottomed flask, DMTMM (3.32 mg dissolved in 0.5 mL Milli-Q water) was added immediately. After 20 min, 5-AF (3.47 mg dissolved in 0.5 mL Milli-Q water) was added. This mixture was then stirred for 2 h at room temperature and purified by 3 k MWCO centrifugal devices to remove the unreacted the 5-AF molecules. The resultant 5-AF-labeled HA solution was stored at 4 °C for further use.

SCC-9 cells were seeded into a 48-well cell culture plate by approximately 1 × 10^4^ cells/well under 100% humidity and then cultured at 37 °C in an atmosphere containing 5% CO_2_ overnight. Then, some wells of adherent cells were treated through being cultured in culture medium contained CD44 antibody (CD44 Ab) (0.1 µg/mL) for 12 h to block the CD44 receptors. After being rinsed with PBS buffer, the FITC-labeled CD44 antibody (0.1 µg/mL) or 5-AF-labeled HA (0.5 mM) was co-incubated with the unblocked or blocked cells for 1 h. After that, the cells were subjected to the confocal laser scanning microscope (Leica, TCS-SP8, Germany) for bright field and fluorescent imaging.

### In vitro cell binding assays

SCC-9 cells were seeded into a 24-well cell culture plate by approximately 2 × 10^4^ cells/well under 100% humidity and then cultured at 37 °C in an atmosphere containing 5% CO_2_ overnight. After being rinsed with PBS buffer, the cells were incubated with the Fe^III^TA@HA nanoprobes solution at a series of Fe^3+^ concentrations including 0, 0.4, and 0.8 mM in same conditions for 4 h at 37 °C. Then the cells were rinsed three times with PBS to remove the unbound free nanoprobes, and further fixed with 4% paraformaldehyde. Then the cells were incubated with Perls Prussian blue stain for Fe detection. The imaging of cells was carried out on an inverted fluorescence microscope (Leica DMI 3000B). Regarding the cell binding assays on CD44 blocked SCC-9 cells, the adherent cells were pretreated through being cultured in culture medium contained CD44 antibody (CD44 Ab) (0.1 µg/mL) for 12 h. Then, following the above procedures for unblocked SCC-9 cells, the binding affinity of the nanoprobes was evaluated.

### Cytotoxicity of Fe^III^TA@HA probes

CCK assays on SCC-9 cells were carried out as follows. Cells were seeded into three 96-well cell culture plates by 5 × 10^3^ cells/well under 100% humidity, and then cultured at 37 °C in an atmosphere containing 5% CO_2_ for 24 h. Thereafter, the nanoprobes with a series of different concentrations were added to three plates and incubated with the cells for 24 h at 37 °C, in which two plates were also introduced the AP (50 µM) and DFO (100 µM) in each well, respectively. Subsequently, the supernatant containing the nanoparticles was decanted. After that, 100 µL culture medium contained 10 µL CCK-8 was added to each well and incubated for another 3 h at 37 °C. Finally, the optical density of each well at 450 nm was recorded on a microplate reader (Thermo, Multiskan GO). Regarding the CD44 blocked SCC-9 cells, the adherent cells were pretreated through being cultured in culture medium contained CD44 Ab (0.1 µg/mL) for 12 h. Then, following the above procedures for unblocked SCC-9 cells, the CCK assays of the nanoprobes was evaluated.

### Detection of intracellular ROS in vitro

SCC-9 cells were seeded into 48-well cell culture plate by ~ 1 × 10^4^ cells/well under 100% humidity, and then cultured at 37 °C in an atmosphere containing 5% CO_2_ for 12 h. Subsequently, the SCC-9 cells were treated with nanoprobes (0.2 mM, with respect to Fe) and PBS in the presence/absence of DFO (100 µM)/AP (50 µM), respectively. Free nanoprobes were removed by washing the cells twice with DMEM after 3 h co-incubation. The cells were stained by DCFH-DA (1 mM) at 37 °C for 40 min and rinsed by PBS twice, and further fixed with 4% paraformaldehyde for 20 min. After nucleus staining by DAPI, the fluorescence images were captured on a fluorescence microscope (Leica DMI 3000B). Regarding the CD44 blocked SCC-9 cells, the adherent cells were pretreated through being cultured in culture medium contained CD44 Ab (0.1 µg/mL) for 12 h. Then, following the above procedures for unblocked SCC-9 cells, the ROS generation was evaluated.

### Evaluation of intracellular GSH depletion of nanoprobes

SCC-9 cells were seeded into a 6-well cell culture plates by ~ 1 × 10^5^ cells/well under 100% humidity, and then cultured at 37 °C in an atmosphere containing 5% CO_2_ for 12 h. Regarding the CD44 blocked SCC-9 cells, the adherent cells were pretreated through being cultured in culture medium contained CD44 Ab (0.1 µg/mL) for 12 h. After co-incubation with a series of Fe^III^TA@HA nanoprobes for 8 h, the cells were harvested and lysed. The resultant lysates were centrifuged (10,000 r/min, 15 min) and the supernatant was used for GSH detection. In detail, 400 µL of the supernatant was added to 100 µL of DTNB (0.75 mM). Then the GSH concentration of each sample was recorded through measuring the absorbance at 412 nm on a microplate reader (Thermo, Multiskan GO).

### Evaluation of intracellular GPX4 levels

SCC-9 cells were seeded into a 24-well cell culture plate by 1 × 10^5^ cells/well under 100% humidity, and then cultured at 37 °C in an atmosphere containing 5% CO_2_ for 12 h. Regarding the CD44 blocked SCC-9 cells, the adherent cells were pretreated through being cultured in culture medium contained CD44 Ab (0.1 µg/mL) for 12 h. After co-incubation with Fe^III^TA@HA nanoprobes (0.5 mM with respect to Fe) for 6 h, the cells were harvested and rinsed. After three cycles of freezing and thawing, the resultant lysates were centrifuged (3000 r/min, 20 min) and the supernatant was subjected to GPX4 ELISA kit (Shanghai Enzyme-linked Biotechnology Co., Ltd.). The GSH levels of each sample were finally recorded through measuring the absorbance at 450 nm on a microplate reader (Thermo, Multiskan GO).

### Photothermal ablation of cancer cells

SCC-9 cells were seeded into a 48-well cell culture plate by ~ 1 × 10^4^ cells/well, and cultured at 37 °C in an atmosphere containing 5% CO_2_ for 24 h. Fe^III^TA@HA nanoprobes were added at the concentrations of 0 and 0.1 mM (with respect to Fe^3+^), and incubated for 4 h before exposed to the irradiation of 650 nm laser with a power density of 1 W·cm^− 2^ for 10 min.

In order to differentiate the viable cells from the dead cells after in vitro photothermal treatment, the 3′,6′-Di(O-acetyl)-4′,5′-bis[N,N-bis(carboxymethyl) aminomethyl] fluorescein, tetraacetoxymethyl ester (calcein-AM)/propidium iodide (PI) staining reagents were adopted to stain the viable cells green (λ_ex_ = 490 nm, λ_em_ = 515 nm) and dead cells red (λ_ex_ = 535 nm, λ_em_ = 617 nm), respectively. Specifically, 100 µL 20 mM of calcein-AM and PI solution were added after the removal of the culture medium and rinsing of the disks. After 30 min of incubation, the staining solution was removed and cells were rinsed by PBS twice for observation with fluorescence microscope (Leica DMI 3000B).

### Animal tumor model

The mice superficial tumor models were established upon subcutaneous inoculation of SCC-9 cells (~ 5 × 10^6^) into 4 weeks old female BALB/c nude mice at right armpit. The tumor imaging studies were carried out 5–7 d after the inoculation of tumor cells.

### MR imaging of tumor in vivo

The MR images were acquired on a 7.0 T animal MRI instrument (Bruker BioSpec70/20USR). BALB/c nude mice bearing SCC-9 superficial tumor xenografts were anesthetized and then the nanoprobe (50 µmol Fe^3+^ per kilogram body weight) or normal saline solution of Gd-DTPA (50 µmol Gd^3+^ per kilogram body weight) was intravenously injected through tail vein. As for the CD44 blocked tumor model, the solid tumor of mice were pretreated with the PBS solution of CD44 antibody for 4 h through intratumoral injection (0.5 mg/kg body weight). *T*_1_-weighted images were acquired pre- and at different time points post-injection. The mice were firstly anesthetized with 2% isoflurane, and the anaesthesia was then maintained with 1.5% isoflurane delivered *via* a nose cone during the imaging sessions. The tumor signal intensities at different time points were quantitatively analyzed through the average *R*_1_ values of tumor areas recorded through the *T*_1_map sequence of MRI. The relative *R*_1_ (Rel. *R*_1_) values of tumors were calculated with reference to the initial *R*_1_ of tumor area recorded prior to the contrast enhancement.

The detailed parameters for MR studies were set as follows:

*T*_1_-weighted imaging: TE = 5.08 ms; TR = 347 ms; FOV = 35 × 35 mm^2^, slice thickness = 1 mm.

*T*_1_map: TE = 5.90 ms, TR = 3000, 1500, 1000, 500, 458.744 ms, FoV = 35 mm × 35 mm, slice thickness = 1 mm.

### Treatment of tumors

20 tumor-bearing BALB/c nude mice with an average tumor volume of ~ 25 mm^3^ were randomly allocated into 5 groups (n = 4). These five groups of mice were administrated with (1) 1× PBS solution of Fe^III^TA@HA nanoprobes (50 µmol Fe^3+^ per kilogram body weight) with 650 nm NIR laser irradiation (Hi-Tech Optoelectronics Co., Ltd. Beijing, China) for 10 min (1 W·cm^− 2^) at 1–2 h post-injection; (2) 1× PBS solution of Fe^III^TA@HA nanoprobes (50 µmol Fe^3+^ per kilogram body weight) without laser irradiation; (3) the same injection volume of 1× PBS solution with 650 nm NIR laser irradiation (Hi-Tech Optoelectronics Co., Ltd. Beijing, China) for 10 min (1 W·cm^− 2^) at 1–2 h post-injection; (4) the same injection volume of 1× PBS solution without laser irradiation; (5) normal saline solution of cisplatin (10 mg per kg body weight), respectively. The tumor size was measured every day and the volume was calculated according to V = (a × b^2^)/2, where a and b represent the length and width of the tumor. Relative tumor volumes were calculated with reference to the initial volume recorded prior to the treatment. The weight of each mouse was also measured every day as well.

### Histopathological analysis of tumor tissues

One representative tumor-bearing BALB/c nude mice were sacrificed 4 h after probe injection, the tumor tissue was extracted. After being embedded into paraffin, the fixed tissues were cut into 4 μm slices, and stained with H&E and Prussian blue for histopathological analysis. One of the tumor slice was also subjected to the immunohistochemistry analysis of CD44 staining.

After 10 days of treatments, the tumor tissues of all groups of mice were extracted for histopathological, immunohistochemistry and immunofluorescence analysis. After being embedded into paraffin, the fixed tumor tissues were sliced. The adjacent tumor slices were subjected to H&E, PCNA, Ki 67 and Caspase 3 staining for studying the proliferation and death of tumor cells with microscopy. The tumor slices were also subjected to CD4/CD8 and TNF-α/IFN-γ staining for the tumor immune studies.

### Biosafety evaluation

The major organs of the above tumor-bearing mice sacrificed at 4 h post-injection and another mice sacrificed at 15 d post-injection were extracted. After being embedded into paraffin, the fixed tissues were cut into 4 μm slices, and stain with H&E and Prussian blue for histopathological analysis.

Three healthy BALB/c mice were intravenously injected with nanoprobes with the dose of 50 µmol Fe^3+^ per kilogram body weight (n = 3). The *R*_1_ values of liver, spleen, and kidney regions were recorded before and at different time points after injection through *T*_1_map sequence.

The detailed parameters of *T*_1_map sequence: TE = 5.90 ms, TR = 3000, 1500, 1000, 500, 458.744 ms, FoV = 35 mm × 35 mm, slice thickness = 1 mm.

### Statistical analysis

Data are shown as the mean ± standard deviation, as indicated in the figure captions. Statistical differences among groups were determined *via* one-way analysis of variance (ANOVA) and Tukey’s multiple comparisons test. A p value < 0.05 was regarded as statistically significant. Both tests were carried out using GraphPad Prism 5.0 software.

## Results and discussion

### Synthesis and characterization of Fe^III^TA@HA nanoprobe

**Fig. 1:**
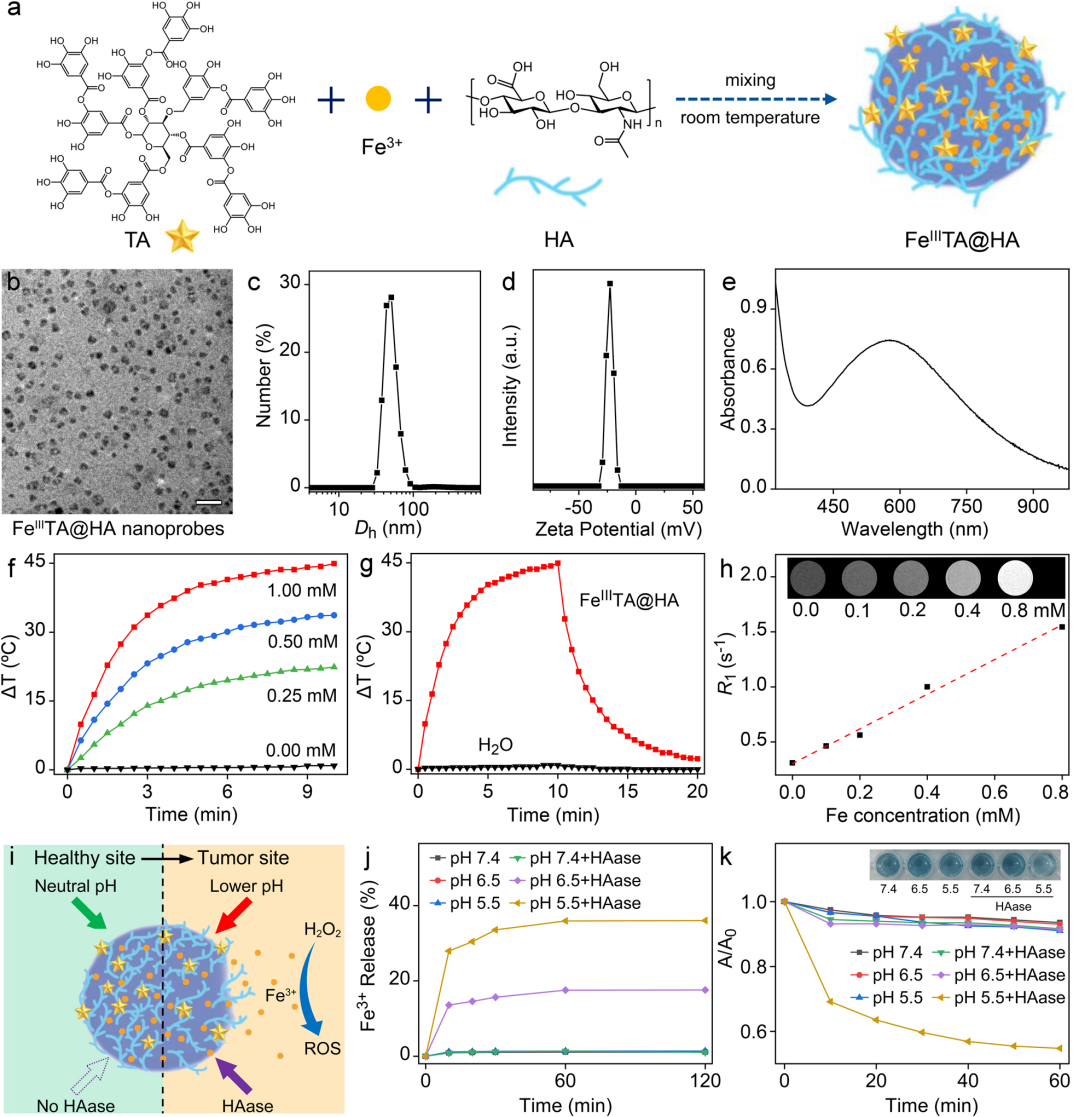
**a** Schematic illustration of Fe^III^TA@HA nanoprobe construction. **b** TEM image of nanoprobes. (The embedded scale bar corresponds to 100 nm). **c** Hydrodynamic size distribution, **d** Zeta potential profiles, and **e** Vis–NIR absorbance spectra of the nanoprobes (0.05 mM of Fe^3+^). **f** Temperature variation of aqueous solutions containing nanoprobes with different Fe concentrations under the irradiation of 650 nm laser (1 W·cm^−2^). **g** The temperature profile of the nanoprobes aqueous solution with the Fe concentration of 1.00 mM irradiated with 650 nm laser, followed by natural cooling after laser was turned off. **h** *T*_1_-weighted MR images of nanoprobe aqueous solutions (inset), together with the linear regression fitting of the *R*_1_ values. **i** Schematic illustration of the dual-stimuli triggered peroxidase-like activity of nanoprobe. **j** Fe^3+^ release kinetics of nanoprobes recorded at different conditions. **k** Normalized absorbance of MB after adding nanoprobe and H_2_O_2_ under different conditions. Inset: Photographs for showing the corresponding visual color changes of MB solutions

Due to the strong coordination reaction between Fe^3+^ and catechol groups of TA, the Fe^III^TA complex with dark purple color could quickly form in aqueous solution (Additional file 1: Fig. S1a). However, the rather low aqueous dispersibility and colloidal stability of these Fe^III^TA complexes will lead to the fast aggregation and precipitation of them, which largely hampers their applications in biomedical fields (Additional file 1: Fig. S1b). Inspired by the bio-mineralization process, HA (*M*_w_ ~8000), a kind of macromolecule, was introduced as the three-dimensional matrixes to control the nucleation and enhance the colloidal stability of Fe^III^TA in water solution, meanwhile endowing the complexes with tumor targeting ability (Fig. 1a). As shown in Fig. S1, through the control of HA, the aggregation of Fe^III^TA complex could be well inhibited, and the stability of resultant complexes in water was significantly improved. To confirm the nano-structure of the complexes, the morphology and the size distribution of them was studied by using transmission electron microscopy (TEM). A uniform nanoprobe with the diameter of 22.5 ± 3.7 nm in average was successfully prepared (Fig. 1b and Additional file 1: Fig. S2). The hydrodynamic diameter (*D*_h_) of this nanoprobe was further investigated with dynamic light scattering (DLS) analysis. As shown in Fig. 1c, the DLS profile presented a single peak, and the average *D*_h_ of Fe^III^TA@HA nanoprobe in aqueous solution was 50.7 nm, further indicating that due to the present of HA, the nucleation and growth processes of nanoprobes took place in a controlled manner, and no large aggregates or precipitates formed. In addition, the zeta potential of Fe^III^TA@HA in aqueous solution was − 22.8 mV (Fig. 1d), suggesting that the HA residues are on the outer surface of the nanoprobes to better realize their targeting ability.

According to our previous publications, Fe^3+^-polyphenol complexes normally show a wide absorption band in both visible and NIR region due to ligand-metal charge transfer (LMCT) effect [38]. This kind of charge transfer transition is orbital- and spin-allowed, which becomes preferable in comparison with *d-d* electronic transition as the latter is parity forbidden. Therefore, the Fe^3+^-polyphenol complexes often exhibits large extinction coefficients. As shown in Fig. 1e, the corresponding Vis–NIR absorption spectra of the Fe^III^TA@HA nanoprobes displayed a wide absorption band from 400 to 900 nm, with a characteristic peak centered at approximately 580 nm. Based on this wide absorption band, Fe^III^TA@HA nanoprobe is promising to serve as a photothermal therapy agent. Based on this hypothesis, the photothermal conversion performance of nanoprobes has been evaluated. The Fe^III^TA@HA nanoprobes with different Fe^3+^ concentrations including 0, 0.25, 0.50 and 1.00 mM were exposed to 650 nm NIR laser with the power density range of 1.0 W·cm^−2^, respectively. The temperature of each solution was recorded for 10 min under continuous laser irradiation until the solution reached a steady temperature (Fig. 1f). Besides, the temperature changes of Fe^III^TA@HA nanoprobes solution with 1.00 mM Fe concentration exposed to laser with different power density including 0.5, 1.0, and 1.5 W·cm^−2^ were also studied through the same procedure (Additional file 1: Fig. S3). As a result, the temperature difference (ΔT) drastically ascended with the increasing particle concentration or the power density of laser. Typically, the ΔT of the Fe^III^TA@HA solution with Fe^3+^ concentration of 1.00 mM could increase by 44.9 °C, after irradiation for 10 min by 650 nm laser with the power density of 1 W·cm^−2^. By contrast, the ΔT of pure water was only 0.9 °C under the same conditions. This result indicated that the nanoprobes possess outstanding photothermal conversion ability.

The photothermal conversion efficiency of Fe^III^TA@HA nanoprobes was further calculated. As the laser was switched off, the relationship between time (t) and the temperature of probe solution (T) could be expressed as [39]:

1 $$t=-{\tau }_{s}{ln}\frac{T-{T}_{surr}}{{T}_{max}-{T}_{surr}}$$

where *τ*_s_ is the system time constant, T_surr_ is the ambient temperature of the surrounding environment, and T_max_ is the equilibrium temperature after laser irradiation. For the sake of simplicity, a dimensionless term θ can be defined, and the Eq. 1 can be simplified:

$${\uptheta }=\frac{T-{T}_{surr}}{{T}_{max}-{T}_{surr}}$$

2 $$t=-{\tau }_{s}{ln}\theta$$

According the heating/cooling profile of the nanoprobe solution (Fig. 1g), *τ*_s_ can be obtained as the slope of the linear regression of the experimental data based on *Eq. 2* (Additional file 1: Fig. S4). Accordingly, the photothermal conversion efficiency of Fe^III^TA@HA nanoprobes can be calculated to be 14.8% under the irradiation of 650 nm laser (the details are provided in Additional file 1), which is comparable to those reported nanomaterials with excellent photothermal ability [40].

Apart from the photothermal efficiency, the MRI properties of Fe^III^TA@HA nanoprobe were also investigated. Owing to the 5 unpaired d-electrons of paramagnetic Fe^3+^ ions, the current nanoprobe is expected to be the *T*_1_ contrast agent for MRI. The MRI performance of the Fe^III^TA@HA was therefore measured on a 7.0 T MRI scanner. As shown in the inset of Fig. 1h, Fe^III^TA@HA nanoprobes exhibited a strong *T*_1_ contrast enhancement effect even under the low Fe concentrations, which brighten the aqueous solution in the *T*_1_-weighted imaging. By linear regression fitting of the longitudinal relaxation rate (*R*_1_) of probe solution with different Fe concentrations, the longitudinal molar relaxivity (*r*_1_) of nanoprobes was extracted as 1.58 mM^− 1^ s^− 1^ (Fig. 1h). On the other hand, the *T*_2_ contrast enhancement ability was also be measured. As shown in Fig. S5, the transverse molar relaxivity (*r*_2_) of nanoprobe was calculated as 4.50 mM^− 1^ s^− 1^ according to the slope of regression curve. Accordingly, the *r*_2_/*r*_1_ ratio of the Fe^III^TA@HA nanoprobe was calculated as 2.85, which is rather low. Therefore, the high *r*_1_ as well as low *r*_2_/*r*_1_ ratio indicated that the as-developed nanoprobe can serve as an ideal candidate for *T*_1_ contrast agents for MRI [41].

### Dual-stimuli triggered Fe^3+^ release and catalytic activity of Fe^III^TA@HA nanoprobes

According to the design concept, the stability of Fe^3+^ ions in nanoprobes mainly depends on two aspects. On one hand, the HA molecules on the surface of nanoprobes can be partly degraded into low-molecular-weight fragments by the HAase. On the other hand, under the lower pH, the phenolic hydroxyl groups of TA molecules will be protonated, causing the dissociation of Fe^III^-TA complexes, and further leading to the release of Fe^3+^. Therefore, it is expected that the nanoprobes will be stable either in neutral environment or in the absence of HAase. In contrast, the Fe^3+^ ions inside the nanoprobes can be released only under the dual stimulation of lower pH and rich HAase (Fig. 1i). To confirm this hypothesis, the Fe^3+^ release behaviors of nanoprobes in different conditions were studied. As shown in Fig. 1j, the Fe^III^TA@HA nanoprobes were quite stable in neutral conditions (pH 7.4) either in the absence or presence of HAase. In contrast, in acidic and rich HAase conditions, the Fe^3+^ release could be detected. Specifically, with the presence of HAase, a fast Fe^3+^ release process could be observed in the first 30 min, in which approximately 15.7% and 33.6% of the total Fe^3+^ were released at tumor microenvironment pH (pH 6.5) and lysosomal pH (pH 5.5), and these released rates still increased gradually, and finally reached 17.6% and 36.1% until the 2 h, respectively. More importantly, neither single stimulation of lower pH nor single stimulation of high HAase could trigger the Fe^3+^ release. Therefore, this result strongly highlighted the design of nanoprobe herein, that is, only the through the dual-stimuli of pH and HAase, the Fe^III^TA@HA nanoprobes could be triggered to release Fe^3+^.

In principle, the released Fe^3+^ ions can catalyze the decomposition of H_2_O_2_ to produce free radicals. On this basis, the Fe^III^TA@HA nanoprobes are promisingly to serve as an anti-cancer agent with peroxidase-like capability.

According to the sensitive dual-stimuli triggered Fe^3+^ released behavior of the nanoprobes, the •OH generation capability of Fe^III^TA@HA nanoprobes was investigated through the methylene blue (MB) degradation. As shown in Fig. 1k, under the presence of HAase, the MB content in aqueous solution decreased by 45.3% under pH 5.5 at 1 h after treated with Fe^III^TA@HA nanoprobe and H_2_O_2_, while it only decreased by 8.3% at pH 7.4. In addition, without HAase, the degradation rates of MB were less than 10% under all these three pH. This result revealed that the current nanoprobes can effectively catalyze the decomposition of H_2_O_2_ to generate •OH under the stimulation of both H^+^ and HAase. The degradation of MB can be also characterized by the color change. As shown in the inset of Fig. 1k, a conspicuous color fading of MB solution can be observed after adding the nanoprobes and H_2_O_2_ at pH 5.5 with the presence of HAase, indicated that at the MB had been largely degraded at this condition.

The above color reaction indicated that the peroxidase-like capability of nanoprobes presents a strong dual-stimuli dependency, which has the similar trend with the Fe^3+^ release. Therefore, it can be reasonably speculated that the peroxidase-like capability of nanoprobes is attributed to the released Fe^3+^. Overall, the Fe^III^TA@HA nanoprobe can act as a dual-stimuli triggered nanozyme with peroxidase-like capability, which exhibits a great potential for tumor therapy.

### Tumor cell binding affinity and anticancer capability of Fe^III^ TA@HA nanoprobes ***in vitro***

**Fig. 2:**
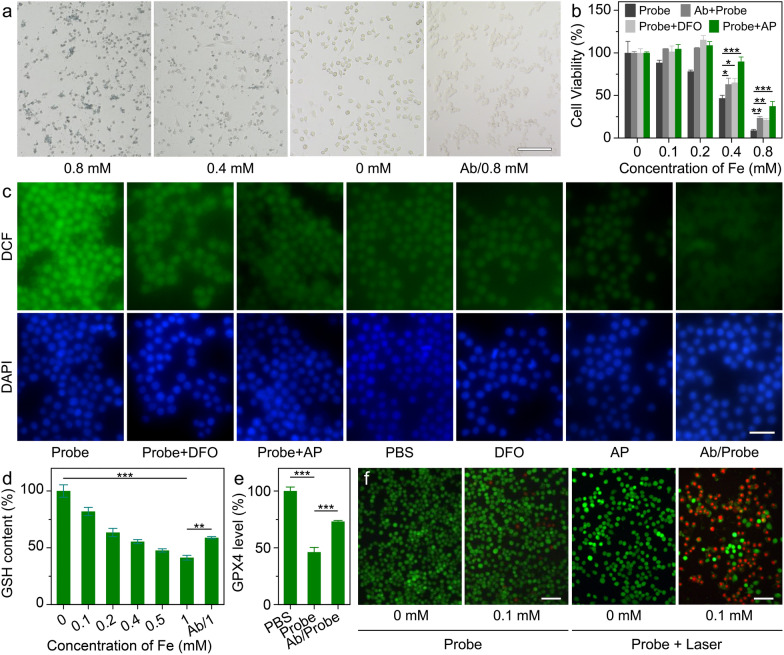
**a** The Perls Prussian stained SCC-9 cells obtained after incubation with Fe^III^TA@HA nanoprobes with different Fe concentrations with or without CD44 Ab. **b** Cell viabilities of SCC-9 cells treated with the Fe^III^TA@HA nanoprobes with different Fe concentrations under different conditions. **c** Fluorescence images of SCC-9 cells treated with different agents, respectively, followed by DAPI staining for showing the cell nuclei and DCFH-DA staining for showing the intracellular ROS. **d** The intracellular GSH contents of SCC-9 cells after co-incubation with nanoprobes with different Fe concentrations with or without CD44 Ab. **e** The intracellular GPX4 levels of SCC-9 cells under different conditions. **f** Cell images acquired after Live-Dead staining for showing the thermal ablation of Fe^III^TA@HA nanoprobes under 650 nm laser irradiation (1.0 W·cm^−2^) for 10 min. Note: the embedded scale bars in the cell images in frame a, c and f correspond to 50 μm, 20 and 50 μm, respectively. Data in frame **b**, **d** and **e** plotted as mean ± standard deviation, n = 3. Statistical significance was determined by one-way ANOVA with a Tukey’s post hoc test (***p < 0.001; **p < 0.01; *p < 0.05)

As a theranostic agent, the tumor-specific targeting ability of nanoprobes is highly desired, whether imaging or therapy. According to the current design principles, the HA molecules of nanoprobes are expected to target the tumor cells through CD44 receptors [21]. To verify this hypothesis, cell line from human tongue SCC (SCC-9 cell line) was used as the model cell. As shown in Fig. 2a, according the Perls Prussian blue staining, the Fe^III^TA@HA nanoprobes exhibited strong cell targeting ability in a concentration dependent manner. In order to verify the targeting specificity of Fe^III^TA@HA nanoprobes, the free CD44 antibody (CD44 Ab) was used as inhibitors to shield the CD44 receptor on SCC-9 cells. As shown in Fig. S6, after CD44 receptor blocking, the binding of FITC-labeled antibodies on blocked SCC-9 cells were significantly inhibited in comparison with unblocked cells (p < 0.0001), indicating that the blocking efficiency of CD44 Ab was very high. More importantly, as displayed in Fig. S7, the binding ability of 5-AF-labeled HA molecules on SCC-9 cells has been also significantly inhibited (p < 0.0001). These results strongly confirmed that the blocked SCC-9 cells can serve as the appropriate negative controls. As shown in Fig. 2a, the uptake of nanoprobes by blocked SCC-9 was significantly inhibited, confirming the good targeting specificity of nanoprobes. This cell uptake difference can be further quantitatively confirmed through the blue signal integral (Additional file 1: Fig. S8). Therefore, CD44-mediated cellular uptake is the main pathway for the nanoprobe to enter SCC-9 cells. As a potential tumor-targeted therapeutic agent, the Fe^III^TA@HA nanoprobes are expected to be specifically toxic to the tumor cells, therefore, the cytotoxicity of the Fe^III^TA@HA nanoprobes was investigated by a standard Cell Counting Kit-8 (CCK-8) assay on SCC-9 cells. The cell viability results given in Fig. 2b revealed that Fe^III^TA@HA nanoprobes exhibited significant cytotoxicity to tumor cells when the concentration of nanoprobe (with respect to Fe^3+^) was as low as 0.4 mM.

Because the CD44-mediated cellular uptake is the main pathway for the nanoprobe to enter SCC-9 cells, it can be reasonably speculated that the blocking of CD44 receptors can reduce the cytotoxicity of the current nanoprobes. To confirm this hypothesis, the CCK-8 assay was also performed on CD44 Ab-pretreated SCC-9 cells. As expectation, compared with normal SCC-9 cells, the viability of CD44 Ab-pretreated SCC-9 cells significantly enhanced (p < 0.05 and p < 0.01 when Fe concentration was 0.4 mM and 0.8 mM, respectively). This result indicated that CD44-mediated cellular uptake is one of the prerequisites for the nanoprobe to exert therapeutic effect.

Considering the peroxidase-like capability of nanoprobes, it can be speculated that the anti-tumor efficacy should be mainly attributed to the released Fe^3+^ under the stimulation of both the HAase secreted by the tumor cells and the lower pH inside the cell lysosomes. To verify this hypothesis, the HAase inhibitor, apigenin (AP), and a type of Fe^3+^ chelator, deferoxamine (DFO), were introduced into the CCK-8 assays, respectively. As a result, the cytotoxicity of nanoprobes could be remarkably decreased (p < 0.001 when the Fe concentration was 0.4 mM and 0.8 mM, respectively) when the tumor cells were co-treated with AP to suppress the secretion of HAase. In addition, DFO can also prevent the nanoprobes to kill cancer cells (p < 0.05 and p < 0.01 when Fe concentration was 0.4 mM and 0.8 mM, respectively). These two results strongly confirmed the aforementioned hypothesis, and further suggested that only the simultaneous stimulation of H^+^ and HAase can trigger the antitumor activity of nanoprobes, highlighting the sensitivity of nanoprobes in cell level.

Additionally, on the basis of the peroxidase-like capability of nanoprobes, the nanoprobes are expected to induce the accumulation of intracellular ROS, and lead to the ferroptosis of tumor cells. Therefore, the excessive ROS of tumor cells was analyzed through DCFH-DA staining (Fig. 2c). Contrasting to the control groups, the strong intracellular green fluorescent signal can be clearly detected after treating with nanoprobes, revealing that the generation and accumulation of excessive ROS. In contrast, after the CD44 receptors were blocked, the nanoprobe-induced intracellular ROS accumulation was dramatically suppressed, which was characterized by the rather weak green signal that was comparable with the normal tumor cells. In addition, the introduction of AP and DFO can also inhibit the nanoprobe-induced ROS accumulation to vary degrees, indicating that the ROS accumulation is mainly attributed to the dual-stimuli responsive Fe^3+^ release of cellular-ingested nanoprobes.

As known, in comparison with the normal cells, the tumor cells with innate oxidative stress are more susceptible to the oxidative damage. However, the generated ROS could also be scavenged by the over-expressed GSH, leading to the limited efficiency of ROS-mediated antitumor therapy. In our design concept, the nanoprobes can not only induce the ROS accumulation, but also consume GSH molecules simultaneously, because the released Fe^3+^ can oxidize the GSH to glutathione oxidized (GSSG) [42]. To confirm this property, the contents of intracellular GSH were evaluated after the tumor cells were co-incubated with the nanoprobes. As shown in Fig. 2d, the intracellular GSH levels of tumor cells exhibited a significant decrease in a nanoprobes concentration dependent manner, but this GSH depletion can be limited by the CD44 blocking. Therefore, it can be concluded that after being ingested, the nanoprobes can largely amplify the oxidative stress of tumor cells through inducing the accumulation of excessive ROS and consuming the intracellular GSH as well, which break the redox homeostasis of tumor cells and lead to the cell death.

In our previous studies, the above ROS accumulation and GSH depletion of tumor cells may lead to the ferroptosis [30, 43]. As known, glutathione peroxidase 4 (GPX4) can convert the potentially toxic lipid hydroperoxides (L-OOH) to non-toxic lipid alcohols (L-OH) to protect the cells. Therefore, the down-regulated expression of GPX4 is believed as features of ferroptosis. Accordingly, the GPX4 level of tumor cells was evaluated through enzyme linked immunosorbent assay (ELISA). As shown in Fig. 2e, the expression of GPX4 in tumor cells was significantly down-regulated (P < 0.001) after treating with the current nanoprobes (0.5 mM with respect to Fe), but if the cells were blocked by CD44 Ab, this trend will be largely attenuated. This result indicated that the ferroptosis induced by the ingested nanoprobes is one of the main pathway of the current nanoprobe for killing cancer cells.

Apart from the dual stimuli triggered catalytic anti-tumor effects, the photothermal anti-tumor efficacy of Fe^III^TA@HA nanoprobes was also investigated through Calcein-AM and propidium iodide (PI) co-staining assay. This mixed cell staining can be used to distinguish the live and dead cells. For the PTT laser source, the 650 nm laser was adopted because the wavelength of it is closed to the He-Ne laser (632.8 nm) or Krypton laser (at 647 nm), which have been adopted in clinical trails. As shown in Fig. 2f, most of cells were destroyed after treating with 0.1 mM nanoprobes (with respect to Fe^3+^) and 650 nm laser (1.0 W·cm^−2^, 10 min). In contrast, almost no cells died after treating with only 0.1 mM nanoprobes without laser irradiation. The result suggested that the current nanoprobes can serve as an ideal PTT agent for photothermal ablation of cancer cells.

**Fig. 3:**
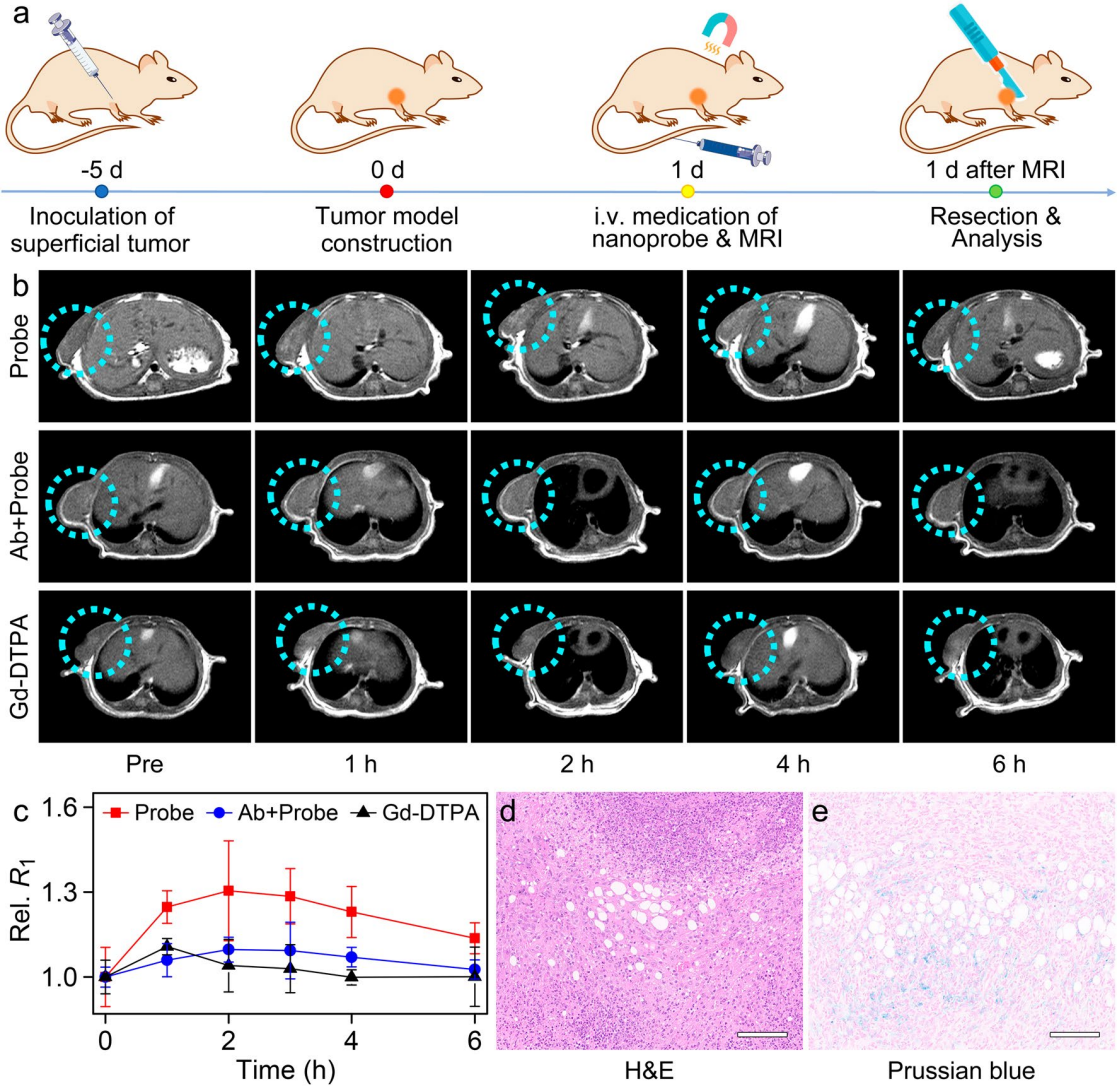
**a** Schematic illustration of SCC-9 subcutaneous tumor establishment, Fe^III^TA@HA nanoprobes administration modalities, and MR imaging. **b** *T*_1_-weighted MR images of subcutaneous tumors or CD44 Ab-treated subcutaneous tumors acquired at different time points of pre- and post-injection of Fe^III^TA@HA nanoprobes and Gd-DTPA contrast agent, respectively, together with **c** corresponding relative *R*_1_ values extracted from the subcutaneous tumor regions at different post-injection time points. **d** H&E and **e** Prussian blue staining of adjacent slices of tumor tissues extracted after imaging. The embedded scale bars correspond to 100 μm

### In vivo MR imaging of the SCC with Fe^III^TA@HA nanoprobes

Based on the in vitro properties of Fe^III^TA@HA nanoprobes, the in vivo tumor targeting and imaging ability was further investigated. The clinical *T*_1_ contrast agent Gd-DTPA was adopted as the control. To better mimic the superficial SCC, BALB/c nude mice bearing SCC-9 subcutaneous tumors were employed as the animal model. Specifically, the SCC-9 tumor cells were inoculated subcutaneously at the right armpit of nude mice. After the successfully establishment of the animal models, the Fe^III^TA@HA nanoprobe or Gd-DTPA was intravenously injected into the tail vein and the mice were then subjected to the MRI. The detailed imaging procedures are given in Fig. 3a. The dose of nanoprobes was determined based on the clinically dosage of Fe_3_O_4_ contrast agent in human MRI, i.e., ~ 50 µmol Fe^3+^ per kg body weight [44]. While the use of Gd-DTPA was also in the range of clinical dosage, i.e. (50 µmol Gd^3+^ per kg body weight) [45]. *T*_1_-weighted MR images of subcutaneous tumor section acquired before and at different time points post-injection are displayed in the Fig. 3b. Accordingly, the tumor region was readily discernible at 1 h after intravenous injection of the probes. Then, the *T*_1_ signals of tumor region reached the intensity maximum from 2 h. Therefore, it was quite evident that the Fe^III^TA@HA nanoprobes exhibited strong tumor targeting ability in vivo, which can provide a good guidance for the following PTT. Thereafter, the enhanced *T*_1_ contrast of tumors began to fade, but can be still distinguished until 6 h post-injection. In order to further confirm the in vivo targeting ability of Fe^III^TA@HA nanoprobes, the subcutaneous tumor of another mouse were pretreated by CD44 Ab to block the CD44 receptors. As shown in the middle row of Fig. 3b, the tumor uptake of nanoprobes were significantly reduced after the pretreatment of CD44 Ab, suggesting that the CD44 specific binding is the predominant reason for nanoprobes to targeting of SCC tumors in vivo.

As for the clinically control, Gd-DTPA can only slightly enhance the contrast of a small part of tumor region at the first 1 h after injection. In addition, this Gd-DTPA-enhanced *T*_1_ signal in tumor area faded up very quickly, and the contrast of tumor had already recovered to the pre-contrast level after 2 h post-injection, as displayed in the last row of Fig. 3b.

The variation of *T*_1_ signals in tumor regions can be also quantitatively characterized through the temporal evolution of the relative local *R*_1_ values of tumor sites pre- and post-contrast. As shown in Fig. 3c, after the administration of nanoprobes, the relative *R*_1_ (Rel. *R*_1_) of the tumor site reached the top at 120 min, which increased by 30.5%, contrasting to only 10.7% recorded from the signal climax of Gd-DTPA contrast agents at 60 min. Overall, the obvious contrast enhancements performance of subcutaneous tumors strongly confirmed that the superficial SCC-targeting ability of the current Fe^III^TA@HA nanoprobes in vivo.

To further validate the active tumor targeting and tumorous accumulation of nanoprobes, one tumor bearing mice were sacrificed at 4 h post-injection of nanoprobes, and solid tumors were extracted for histochemical analysis. The CD44 expression of SCC-9 solid tumor was firstly evaluated. According to the immunohistochemistry result (Additional file 1: Fig. S9), CD44 receptor are significantly over-expressed within tumor, especially the margin region, where the cancer cells proliferate and invade more rapidly. Therefore, the overexpressed CD44 provides enough target sites for the Fe^III^TA@HA nanoprobes to bind with. In addition, two adjacent tumor slices were subjected to hematoxylin-eosin (H&E) and Prussian blue staining for the histopathological analysis and iron assessment. As shown in Fig. 3d and e, within the tumor tissue confirmed by the cell morphology in H&E staining, the Prussian blue signals can be readily observed. This histological analysis implied that a certain amount of probe sdistributed within the tumor at 4 h post-injection, which strongly confirmed the remarkable tumor-targeting specificity of Fe^III^TA@HA nanoprobes.

### Therapeutic efficacy of Fe^III^TA@HA nanoprobes on tumors ***in vivo***

Based on the catalytic and photothermal therapy in cell level and the tumor targeted ability of nanoprobes in vitro and in vivo, the anti-tumor therapeutic efficacy of Fe^III^TA@HA nanoprobes was investigated in vivo. Just like imaging experiments, the BALB/c nude mice bearing SCC-9 subcutaneous tumors was adopted to mimic the superficial SCC. Briefly, 20 tumor-bearing mice with tumor volume ~ 25 mm^3^ were randomly divided into 5 groups (n = 4), which were receiving intravenous injection of PBS, PBS with 650 nm NIR laser irradiation (1 W·cm^−2^, 10 min), first-line chemotherapy drug of SCC (cisplatin), Fe^III^TA@HA nanoprobes, and Fe^III^TA@HA nanoprobes with 650 nm NIR laser irradiation (1 W·cm^−2^, 10 min), respectively. In order to optimize the PTT effect,

**Fig. 4:**
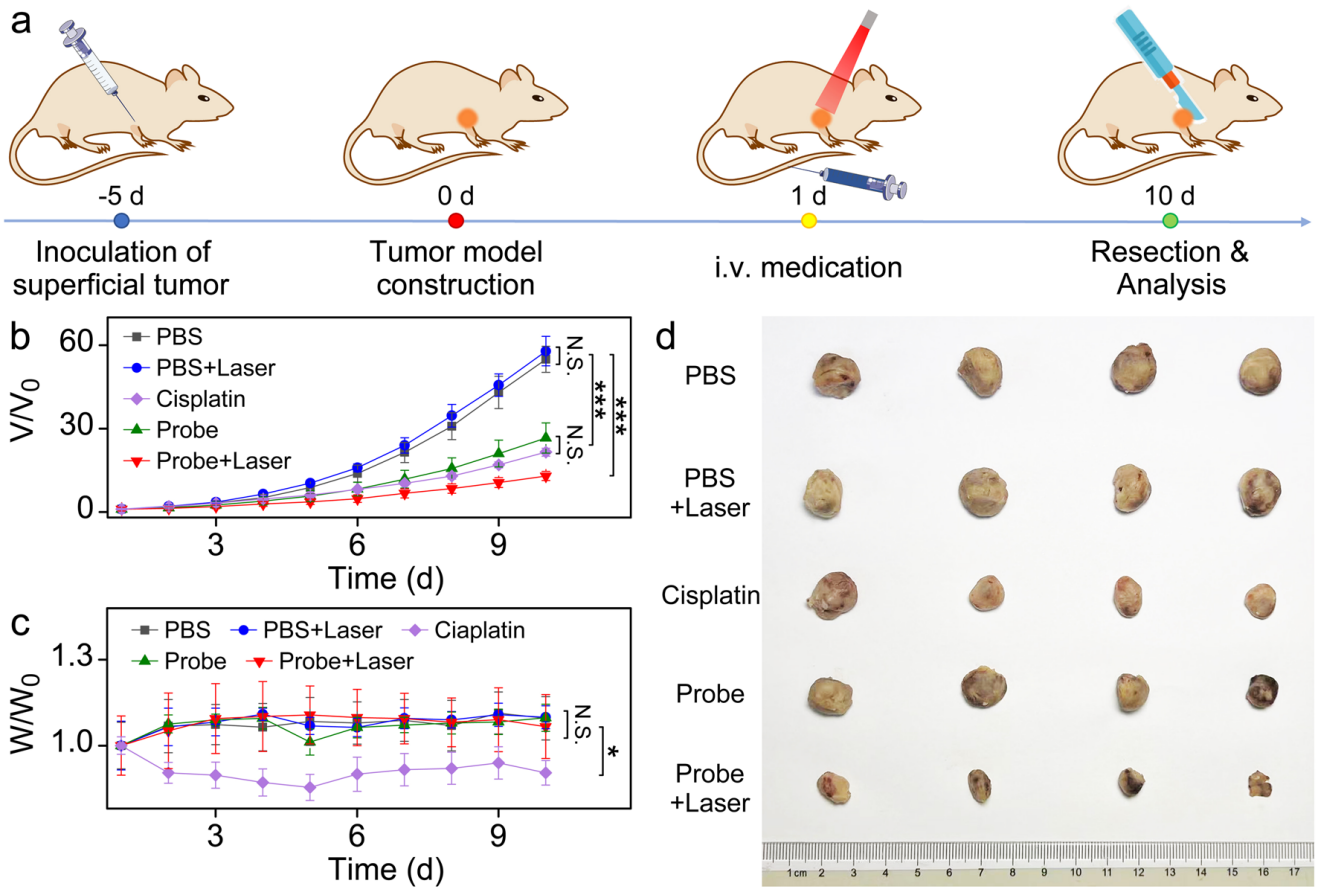
**a** Schematic illustration of SCC-9 subcutaneous tumor establishment, Fe^III^TA@HA nanoprobes administration modalities, and therapeutic approaches. **b** Growth curves of SCC-9 tumors and **c** fluctuation of the body weight of mice in different groups during treatment. **d** Photographs of the dissected tumors from each group after 10 days of treatment. Data in frame **b** and **c** plotted as mean ± standard deviation, n = 4. Statistical significance was determined by one-way ANOVA with a Tukey’s post hoc test (***p < 0.001; *p < 0.05; N.S. Not statistically significant, p > 0.05)

the laser irradiation time point was determined at 1–2 h post-injection according to the MRI results mentioned before. The schematic illustration of the therapy process was shown in Fig. 4a.

In order to quantitatively evaluate the therapy efficacy, the tumor sizes were measured every day during the whole period of treatment (Fig. 4b). Quite remarkably, compared with PBS control group, the probe + laser treatment of tumor can significantly slow the tumor growth. In contrast, without the aid of nanoprobes, 650 nm laser exposure had no significant impact on tumor growth in comparison with mice treated with only PBS. In addition, without laser irradiation, the nanoprobe alone also presented a certain therapeutic efficacy in comparison with PBS group, which was comparable with the single dose of cisplatin treated group. Considering the catalytic therapy capability of nanoprobes confirmed in cell level, this in vivo therapeutic effect of nanoprobes alone can be mainly attributed to the ROS accumulation and GSH depletion induced cell death.

According to the tumor measurement data on the last day of treatment course (10 day post-treatment), the tumor receiving the Fe^III^TA@HA nanoprobes and 650 nm laser irradiation exhibited a smallest volume (12.9-fold of the original) compared with the controls (p < 0.001). In addition, the tumors receiving only the Fe^III^TA@HA nanoprobes treatment displayed a 26.6-fold increase in volume, which is higher than that of laser irradiation groups, but significantly smaller than that of PBS and PBS + laser group with 54.9-fold and 57.8-fold increase (p < 0.001), respectively. In addition, during the treatment process, the body weight of mice from probe + laser and probe alone groups showed the same increasing trend as that of the PBS and PBS + laser groups (p > 0.05). In contrast, cisplatin would significantly lead to the weight loss of mice (p < 0.05), as displayed in Fig. 4c, indicating that it may have some side effects on mice bodies.

To directly evaluate the tumor therapeutic effects of these treatment groups, the mice were sacrificed after 10-day treatment course and then the tumor tissues were harvested. As shown in Fig. 4d, the tumors from mice receiving the Fe^III^TA@HA nanoprobes with PTT were remarkably smaller than that of mice receiving PBS in diameter, and the size relationship of the tumors in different groups ex vivo was consistent with that measured at the last day of treatment in vivo. Therefore, both in vivo and ex vivo results suggested that the current nanoprobe exhibited a significant antitumor efficacy.

Overall, on the basis of the above tumor treatment experiments, the Fe^III^TA@HA nanoprobes could not only prevent the proliferation of tumor cells by themselves, but also served as a powerful PTT agent for tumor physiotherapy. This combined multimodal therapy can undoubtedly pave a new way for the superficial SCC treatment.

**Fig. 5:**
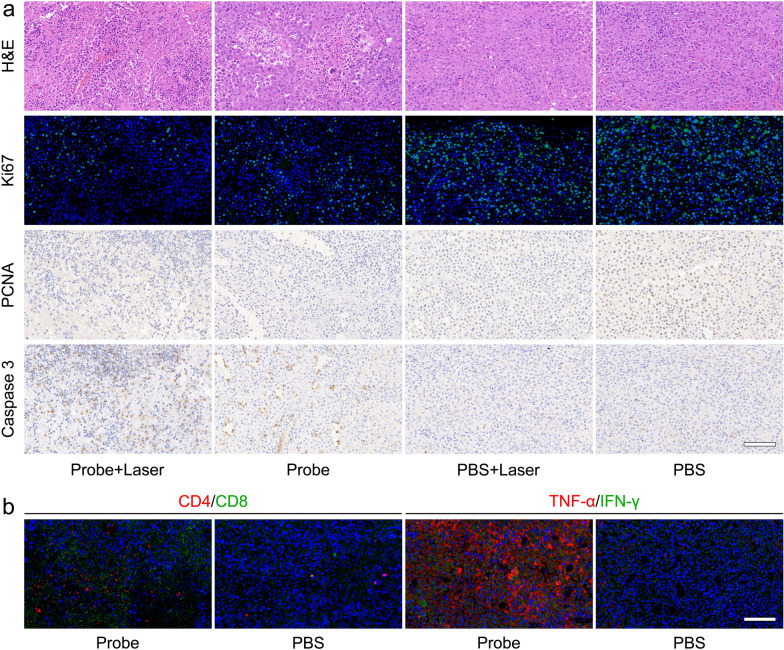
**a** The H&E, Ki 67, PCNA, and Caspase3 staining images of the tumor slices after 10 d treatments. **b** The CD4/CD8 and TNF-α/IFN-γ staining images of the tumor slices after 10 d treatments. The embedded scale bars correspond to 100 μm

To provide much deeper insights into the mechanism of antitumor effect of Fe^III^TA@HA nanoprobes in vivo, the tumor tissue slices from the treatment groups including probe + laser, probe, PBS + laser, and PBS were also subjected for histochemical analysis. According to the H&E staining given in the first row of Fig. 5a, after probe-enhanced PTT of tumor, the cell nuclei were atrophied with the deeper staining, and cell density in the tumor tissue was significantly reduced, which suggested that the PTT can largely destroy the structure of tumor cells. In addition, without laser irradiation, the infiltration of inflammatory cells could be observed in the tumor area after the treatment of probe, suggesting that the probe may spark the immune reaction against tumor. Furthermore, the immunohistochemical and immunofluorescence studies were also carried out to disclose the molecular and cell biological mechanisms of nanoprobe against cancer. As shown in the second and third row of the Fig. 5a, the proliferating cell nuclear antigen (PCNA) and cell cycle-associated protein Ki 67 were barely expressed in the tumor cells after the probe-enhanced PTT in comparison with PBS group, which suggested that the PTT effectively inhibit the proliferation of tumor cells. In addition, both the PCNA and Ki 67 of tumor cells also apparently down-regulated after treating with the nanoprobe alone, which confirmed that the current nanoprobes can continuously prevent the proliferation of tumor cells in a long period after one dose treatment. These results are in coincidence with the.

macroscopic tumor suppressive effect shown in tumor growth curves (Fig. 4b), In addition, through the caspase 3 staining presented in the last row of Fig. 5a, a great percentage of caspase 3-positive cells can be observed after probe-enhanced PTT, indicating that the apoptotic process was occurred in the tumor tissue. Moreover, the caspase 3-positive cells can be also found in the tumor slices of mice treated with the nanoprobes alone, implying that the nanoprobes can also induce the apoptosis of the tumor cells in a long period after medication. Considering the catalytic therapy capability of nanoprobes confirmed in vitro, it can be reasonably speculated that the apoptosis of tumor cells is caused by the oxidative stress. In addition, in comparison with the probe-related treatments, the only laser treatment hardly affects the growth of tumor cells, which is consistent with the tumor growth trend shown in Fig. 4, further highlighting the photothermal ability of the current nanoprobes.

Apart from the above histochemical studies, the recent studies have shown that the HA molecules bound on the tumor cells can guide the lymphocytes to migrate deep into the tumors, thereby enhancing the efficacy of immunotherapy (IT) [46]. Very interestingly, among the different lymphocytes, the CD4^+^ and CD8^+^ T cells have been confirmed to not only suppress the tumor growth through secreting various cytokines, but also enhance ferroptosis-specific lipid peroxidation in tumor cells [47]. If so, these different antitumor pathways will fight against cancer synergistically in the current work. To test this hypothesis, the expression levels of CD4 and CD8 in tumor slices were evaluated. As shown in Fig. 5b, the contents of both CD4 (red) and CD8 (green) was significantly up-regulated within the probe-treated tumor tissue compared with the PBS-treated one, implying that the nanoprobes can recruit a large amount of CD4^+^ helper T lymphocytes (Th) and CD8^+^ cytotoxic T lymphocytes (CTL) deeply into the tumor tissues. In addition, the tumor necrosis factor (TNF)-α and interferon (IFN)-γ, the cytokines that can be released by effector CD8^+^ Th or CD4^+^ CTL to inhibit tumor growth and promote tumor immunity, were also detected through immunofluorescence staining. As a result, both the TNF-α (red) and IFN-γ (green) are appeared to be dramatically up-regulated in the tumor tissue after probe treatment. These results suggested that the antitumor immune response was successfully activated by nanoprobes, and the IT is also one of the pathways of the nanoprobes to fight against tumor cells.

Combined the dual-stimuli triggered Peroxidase-like capability, and the results of in vitro/in vivo experiments, it can be concluded that the Fe^III^TA@HA can quickly eradicate a large part of tumor cells through an MRI-guided instantaneous PTT, and continuously prevent the proliferation of the surviving tumor cells through the long-term hybrid ferroptotsis/apoptosis. Meanwhile, the nanoprobes can also promote the recruitment of immune cells such as CD4^+^ Th and CD8^+^ CTL cells to accumulate in the tumor area to inhibit the tumor growth through the cytokines secretion and ferroptotsis enhancement. These synergistic effects can perform an outstanding tumor treatment efficacy of tumor, especially for the superficial SCC herein.

### In vivo biosafety evaluation of nanoprobes

In the previous work, after intravenous medication, most nano-agents would be recognized by the immune system, and then largely captured by the reticuloendothelial system (RES), such as lung, liver, and spleen [48]. Such undesired retentions potentially cause unpredictable side effects of the body, which has aroused concerns among researchers. To study the clearance of the Fe^III^TA@HA nanoprobes, two representative BALB/c nude mice were sacrificed at 4 h or 15 d after the injection of nanoprobes, and the main organs of them were extracted and cut into slices for histological analysis. According to the Prussian staining, at 4 h post-injection, the probes were only marginally retained in the lung, but had already been cleared in other main organs. By the 15 days, the lungs-trapped probes were also eliminated completely, indicating that the current probes have excellent biosafety without undesired retention in vivo (Additional file 1: Fig. S10). In addition, these main organs of mice were also examined by H&E staining to show the cell morphology for determining whether there was potential organ injury after probe treatment (Additional file 1: Fig. S11). The results further revealed that there were no noticeable inflammation or damage in any major organs induced by the Fe^III^TA@HA probes both at 4 h and 15 d.

Apart from the representative histological analysis, the elimination process of the nanoprobes were further investigated through MR studies. Specifically, three BALB/c mice were administrated with nanoprobes, and the pharmacokinetic behaviors of nanoprobes in liver, kidney and spleen were quantitatively measured by the *R*_1_ values, as displayed in Fig. S12. The *R*_1_ values of liver, spleen, and kidney increased within the 48 h after medication. Nevertheless, the *R*_1_ values of all these organs return to the pre-injected level after 120 h, suggesting that the nanoprobes could be gradually eliminated from the body within several days. All of these results indicated that the Fe^III^TA@HA nanoprobes are rather safe at the present dose level for SCC theranostic applications.

## Conclusion

In summary, a novel pH/HAase dual-stimuli triggered smart nanoprobe Fe^III^TA@HA has been designed and prepared through the biomineralization of Fe^3+^ and polyphenol TA under the control of HA matrix to realize precise theranostics of superficial SCC. With the HA residues on the outer surface, Fe^III^TA@HA nanoprobes can specifically target the CD44 receptors over-expressed on the SCC cells and accumulate in the carcinoma region after intravenously administration. The abundant HAase in carcinoma TME will trigger the degradation of HA molecules, thereby exposing the Fe^III^TA complex. After ingesting by tumor cells *via* CD44 mediated endocytosis, the acidic lysosomal condition will further trigger the protonation of TA molecules, finally leading to the Fe^3+^ release of nanoprobe. Subsequently, these released Fe^3+^ ions can induce a hybrid ferroptosis/apoptosis of tumor cells through peroxidase activity and GSH depletion. In addition, Owing to the outstanding *T*_1_ MRI performance and phototermal conversion efficiency of nanoprobes, the MRI-guided PTT can be also combined to complement the Fe^3+^-induced cancer therapy. Meanwhile, the nanoprobes can also promote the recruitment of immune cells such as CD4^+^ Th and CD8^+^ CTL cells to accumulate in the tumor tissues to inhibit the tumor growth through the cytokines secretion and ferroptotsis enhancement. As a result, through an instantaneous PTT, the current nanoprobes can quickly eradicate a large part of tumor cells, and continuously prevent the proliferation of the surviving tumor cells through the long-term apoptosis/ferroptosis and IT, thereby performing an outstanding tumor treatment efficacy. As for the safety evaluation, the Fe^III^TA@HA nanoprobes can be eliminated from the body gradually within several days, and no obvious adverse side effect was observed through the organ tissue histological analysis, which confirmed the biosafety features of the nanoprobes. We thus believe the current nanoprobes has huge potential in clinical translation in the field of precise diagnosis and intelligent synergistic therapy of superficial SCC. This strategy will promisingly avoid the surgical defects, and reduce the systemic side effect of traditional chemotherapy, paving a new way for the future SCC treatment.

## Supplementary Information

**Additional file 1: Fig. S1.** The photographs of the crystallization of Fe^III^TA complex and biomineralization of Fe^III^TA@HA nanoprobes at 0 and 24 h, for showing the solubility and stability of the crystals. **Fig. S2.** The histogram showing the size distribution profile of Fe^III^TA@HA nanoprobes. **Fig. S3.** Temperature variation of solutions containing Fe^III^TA@HA nanoprobes with the irradiation of 650 nm laser with different power densities. **Fig. S4.** Determination of the system time constant using linear regression of the cooling profile after irradiation of 650 nm laser. **Fig. S5.** The linear regression fitting of the *R*_2_ values of aqueous solutions of Fe^III^TA@HA nanoprobes with different Fe concentrations for extracting the transverse molar relaxivity *r*_2_. **Fig. S6.** The binding of FITC-labeled CD44 Ab on unblocked and blocked SCC-9 cells, together with the quantitative analysis of the fluorescence signals. **Fig. S7.** The binding of 5-AF-labeled HA on unblocked and blocked SCC-9 cells, together with the quantitative analysis of the fluorescence signals. **Fig. S8.** The integrated blue signals of the field of view of the Prussian staining of cells. **Fig. S9.** CD44 staining of the different regions of SCC-9 tumor slice. **Fig. S10.** Prussian staining of tissue slices from major organs of mice treated with Fe^III^TA@HA nanoprobes or PBS for showing the retention of nanoprobes. **Fig. S11.** H&E staining of tissue slices from major organs of mice treated with Fe^III^TA@HA nanoprobes or PBS. **Fig. S12.** Temporal evolution of relative *R*_1_ values of the liver region, spleen region, renal cortex region, and renal pelvis region of mice. Together with the calculation of the photothermal conversion efficiency.

## Data Availability

All data generated or analyzed during this study are included in this article.

## Abbreviations

SCC: Squamous cell carcinoma
MRI: Magnetic resonance imaging
PTT: Photothermal therapy
DMEM: Dulbecco’s modified eagle medium
ELISA: Enzyme linked immunosorbent assay
LMCT: Ligand-to-metal charge transfer
MB: Methylene blue
DFO: Deferoxamine
AP: Apigenin
