# Supplementary material for: Dual-stimuli responsive smart nanoprobe for precise diagnosis and synergistic multi-modalities therapy of superficial squamous cell carcinoma
Source: J Nanobiotechnology. 2023 Jan 3;21:4. doi: 10.1186/s12951-022-01759-1 (PMC9808965; doi:10.1186/s12951-022-01759-1)
Supplement: Supplementary file 1 — Additional file 1: Fig. S1. The photographs of the crystallization of FeIIITA complex and biomineralization of FeIIITA@HA nanoprobes at 0 and 24 h, for showing the solubility and stability of the crystals. Fig. S2. The histogram showing the size distribution profile of FeIIITA@HA nanoprobes. Fig. S3. Temperature variation of solutions containing FeIIITA@HA nanoprobes with the irradiation of 650 nm laser with different power densities. Fig. S4. Determination of the system time constant using linear regression of the cooling profile after irradiation of 650 nm laser. Fig. S5. The linear regression fitting of the R2 values of aqueous solutions of FeIIITA@HA nanoprobes with different Fe concentrations for extracting the transverse molar relaxivity r2. Fig. S6. The binding of FITC-labeled CD44 Ab on unblocked and blocked SCC-9 cells, together with the quantitative analysis of the fluorescence signals. Fig. S7. The binding of 5-AF-labeled HA on unblocked and blocked SCC-9 cells, together with the quantitative analysis of the fluorescence signals. Fig. S8. The integrated blue signals of the field of view of the Prussian staining of cells. Fig. S9. CD44 staining of the different regions of SCC-9 tumor slice. Fig. S10. Prussian staining of tissue slices from major organs of mice treated with FeIIITA@HA nanoprobes or PBS for showing the retention of nanoprobes. Fig. S11. H&E staining of tissue slices from major organs of mice treated with FeIIITA@HA nanoprobes or PBS. Fig. S12. Temporal evolution of relative R1 values of the liver region, spleen region, renal cortex region, and renal pelvis region of mice. Together with the calculation of the photothermal conversion efficiency. [file 12951_2022_1759_MOESM1_ESM.docx]

**Additional Information**

**Dual-Stimuli Responsive Smart Nanoprobe for Precise Diagnosis and Synergistic Multi-modalities Therapy of Superficial Squamous Cell Carcinoma**

*Peisen Zhang^1,2#^, Yingying Cui^2#^,* *Jian Wang^3#^, Junwei Cheng^2^, Lichong Zhu^2^, Chuang Liu^2^, Saisai Yue^2^, Runxin Pang^2^, Jiaoqiong Guan^1^, Bixia Xie^2^, Ni Zhang^4^, Meng Qin^2,4^, Lihong Jing^5^, Yi Hou^2^, Yue Lan^1,^**

^1^Department of Rehabilitation Medicine, Guangzhou First People’s Hospital, School of Medicine, South China University of Technology, Guangzhou, 510180, China

^2^College of Life Science and Technology, Beijing University of Chemical Technology, Beijing, 10029, China.

^3^Department of Head and Neck Surgery, National Cancer Center/National Clinical Research Center for Cancer/Cancer Hospital, Chinese Academy of Medical Sciences, Peking Union Medical College, Beijing, 100021, China.

^4^Department of Psychiatry, and National Chengdu Center for Safety Evaluation of Drugs, West China Hospital, Sichuan University, Chengdu, 610041, China

^5^Key Laboratory of Colloid, Interface and Chemical Thermodynamics, Institute of Chemistry, Chinese Academy of Sciences, Beijing, 100190, China


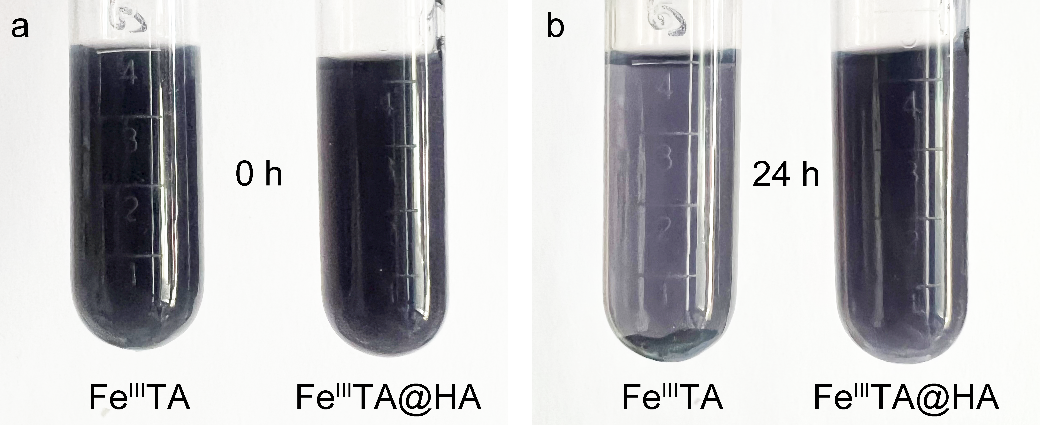


**Fig. S1** Photographs of the crystallization of Fe^III^TA complex and biomineralization of Fe^III^TA@HA nanoprobes at a) 0 h and b) 24 h, for showing the solubility and stability of the crystals.


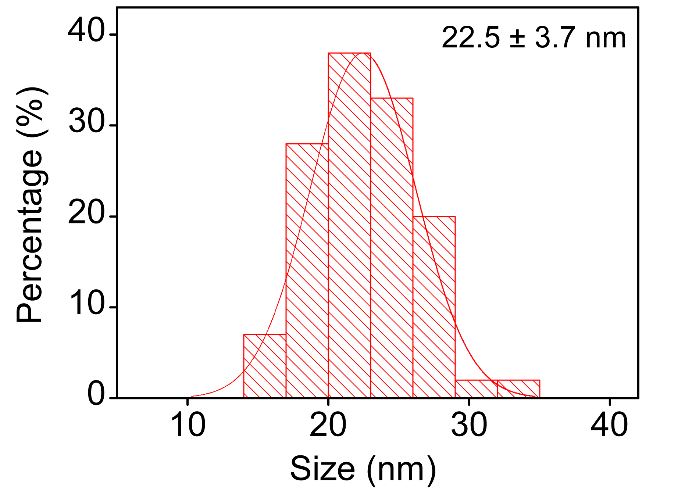


**Fig. S2** The histogram showing the size distribution profile of Fe^III^TA@HA nanoprobes.


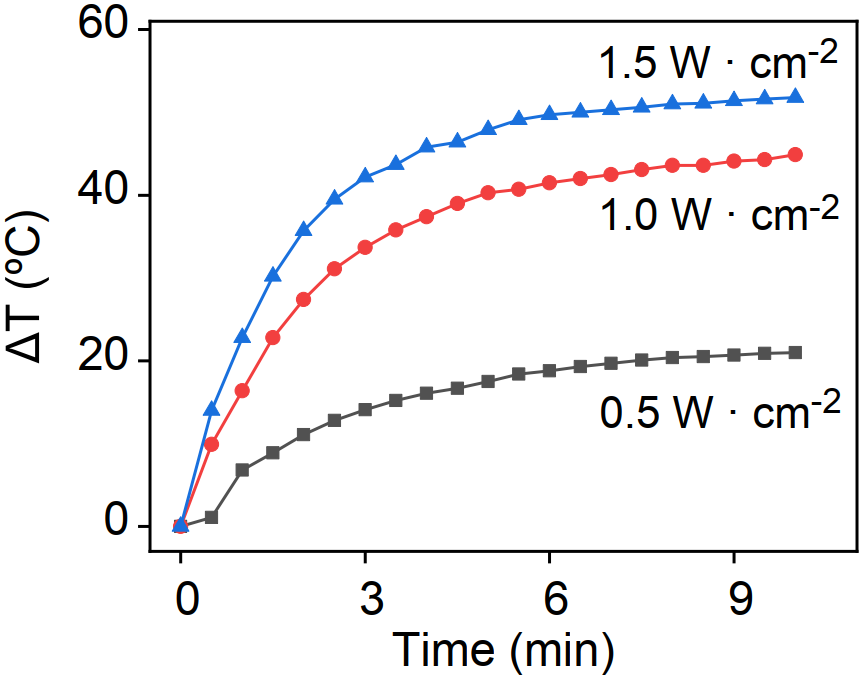


**Fig. S3** Temperature variation of solutions containing Fe^III^TA@HA nanoprobes (1.00 mM equivalent to Fe) with the irradiation of 650 nm laser with different power densities.


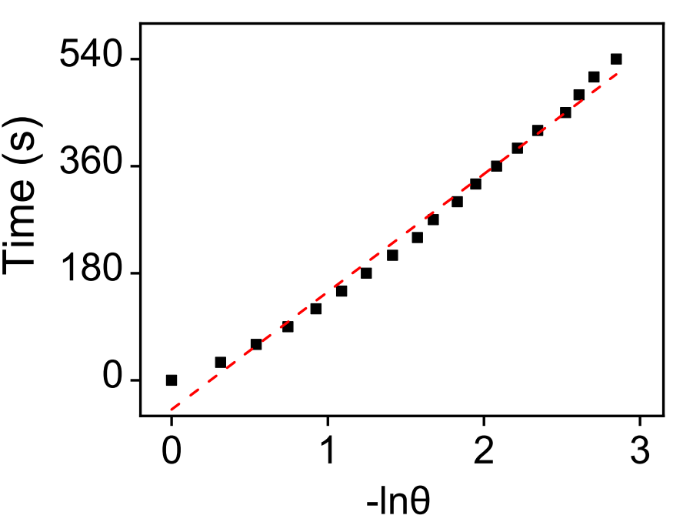


**Fig. S4** Determination of the system time constant using linear regression of the cooling profile after irradiation of 650 nm laser.


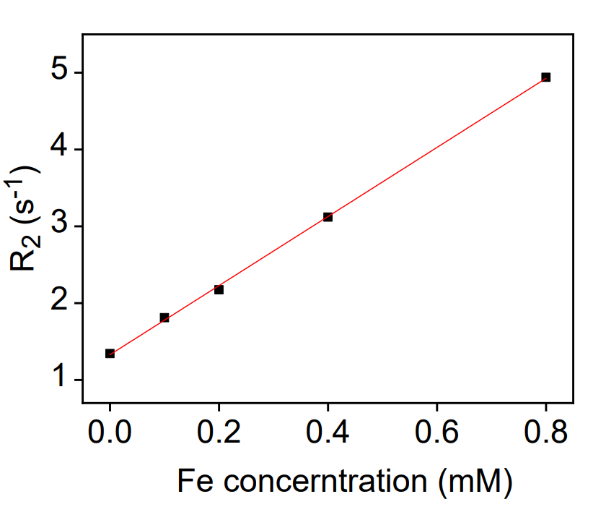


**Fig. S5** The linear regression fitting of the *R*_2_ values of aqueous solutions of Fe^III^TA@HA nanoprobes with different Fe concentrations for extracting the transverse molar relaxivity *r*_2_


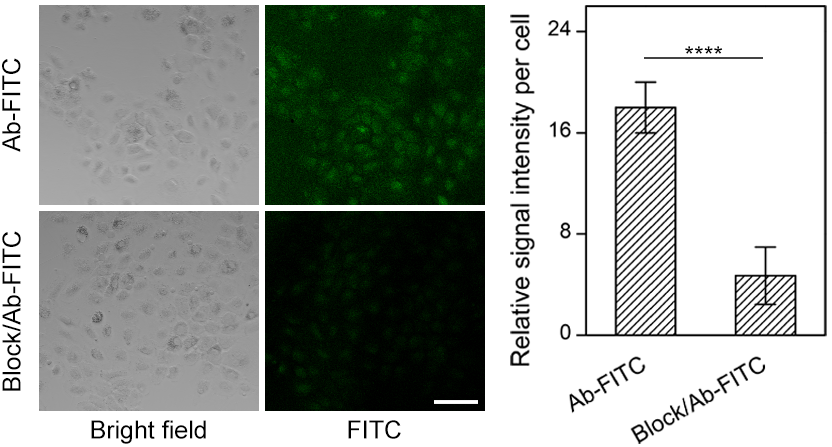


**Fig. S6** The binding of FITC-labeled CD44 Ab on unblocked and blocked SCC-9 cells (left), together with the quantitative analysis of the fluorescence signals (right). The embedded scale bar corresponds to 100 μm. Statistical significance was determined by Student’s t-test. ****p < 0.0001.


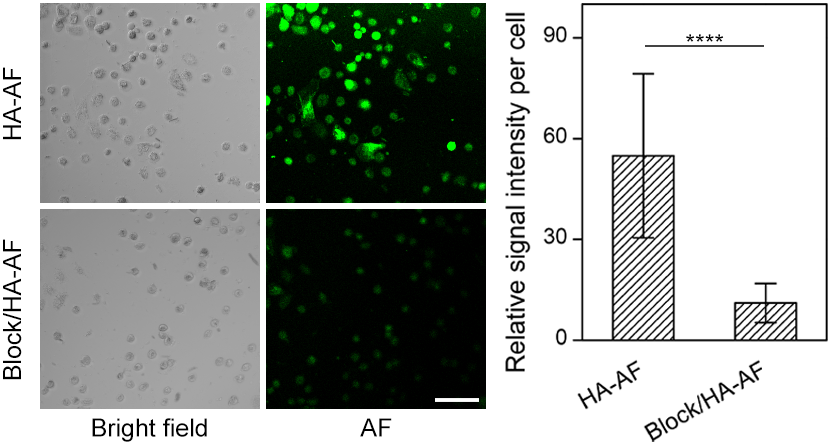


**Fig. S7** The binding of 5-AF-labeled HA on unblocked and blocked SCC-9 cells (left), together with the quantitative analysis of the fluorescence signals (right). The embedded scale bar corresponds to 100 μm. Statistical significance was determined by Student’s t-test. ****p < 0.0001.


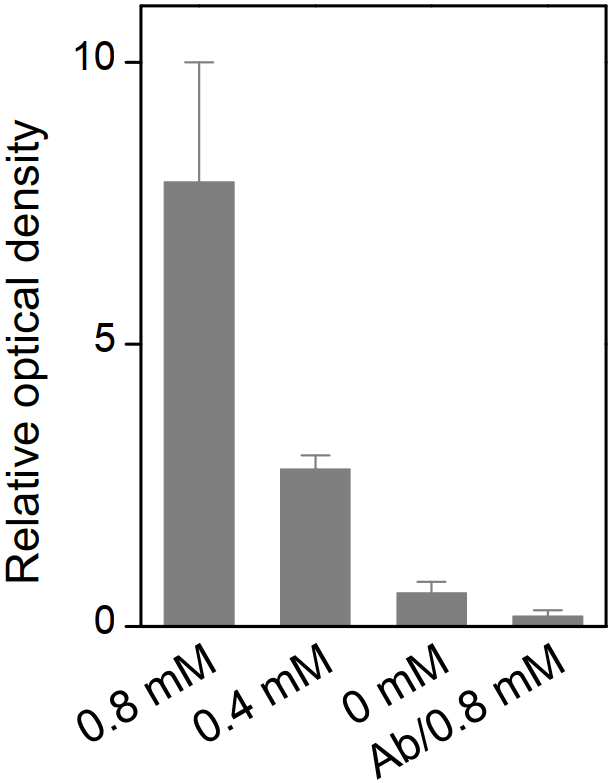


**Fig. S8** The integrated blue signals of the field of view of the Prussian staining of cells.


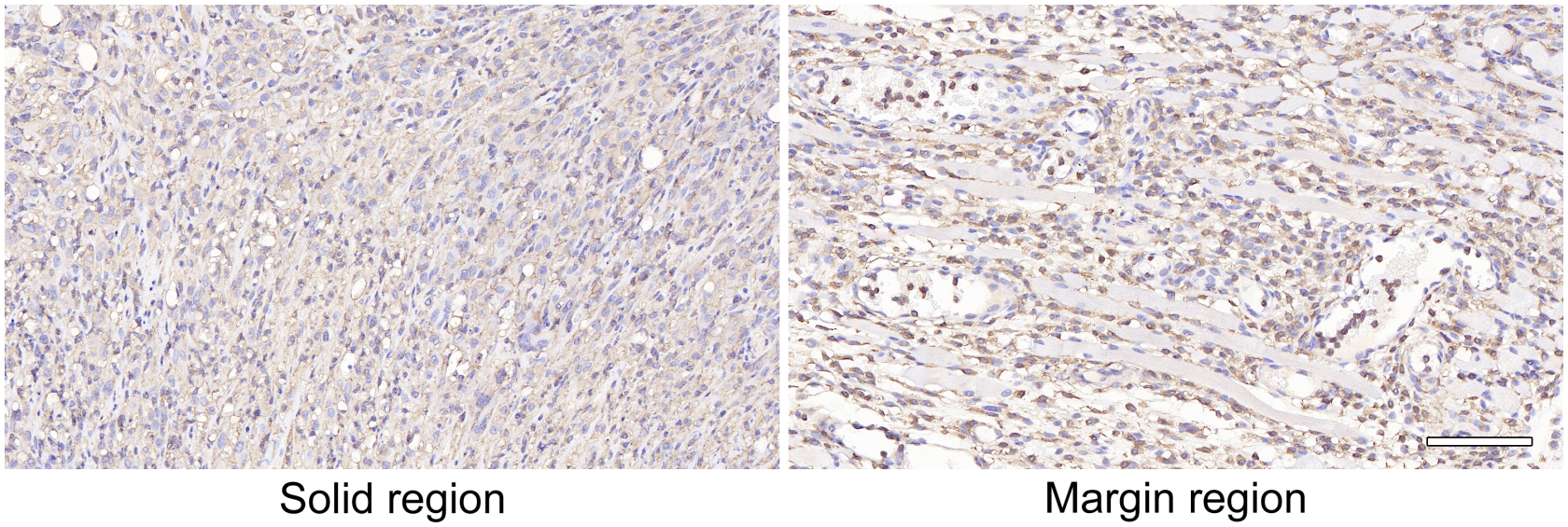


**Fig. S9** CD44 staining of the different regions of SCC-9 tumor slice. The embedded scale bar corresponds to 100 μm.

**
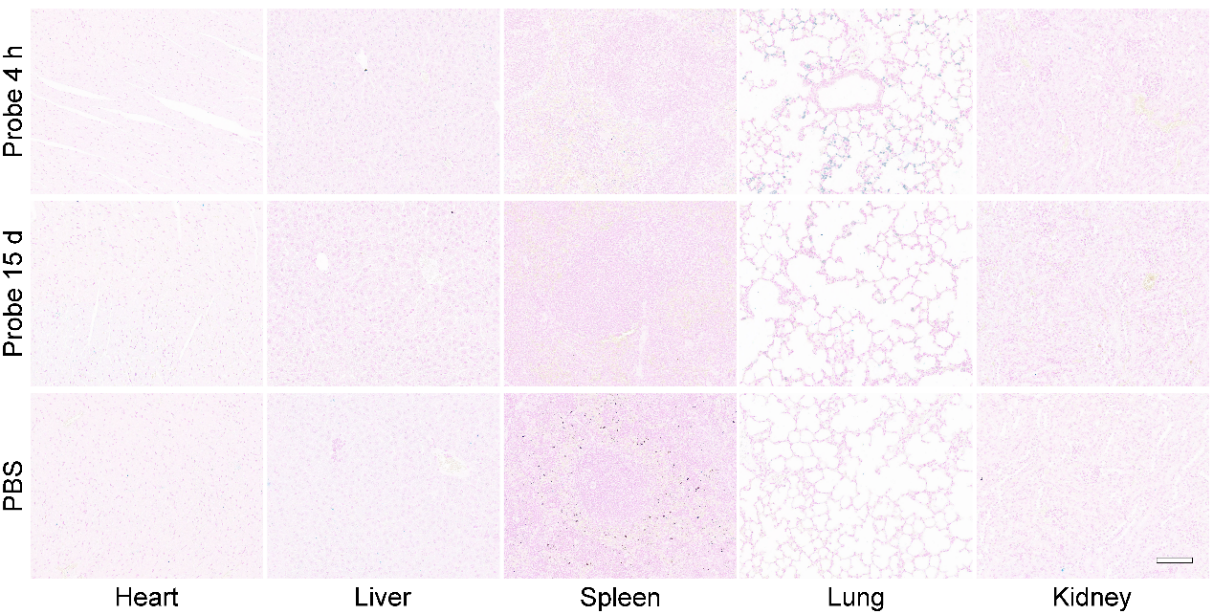
**

**Fig. S10** Prussian staining of tissue slices from major organs of mice treated with Fe^III^TA@HA nanoprobes or PBS for showing the retention of nanoprobes. The embedded scale bar corresponds to 100 μm.


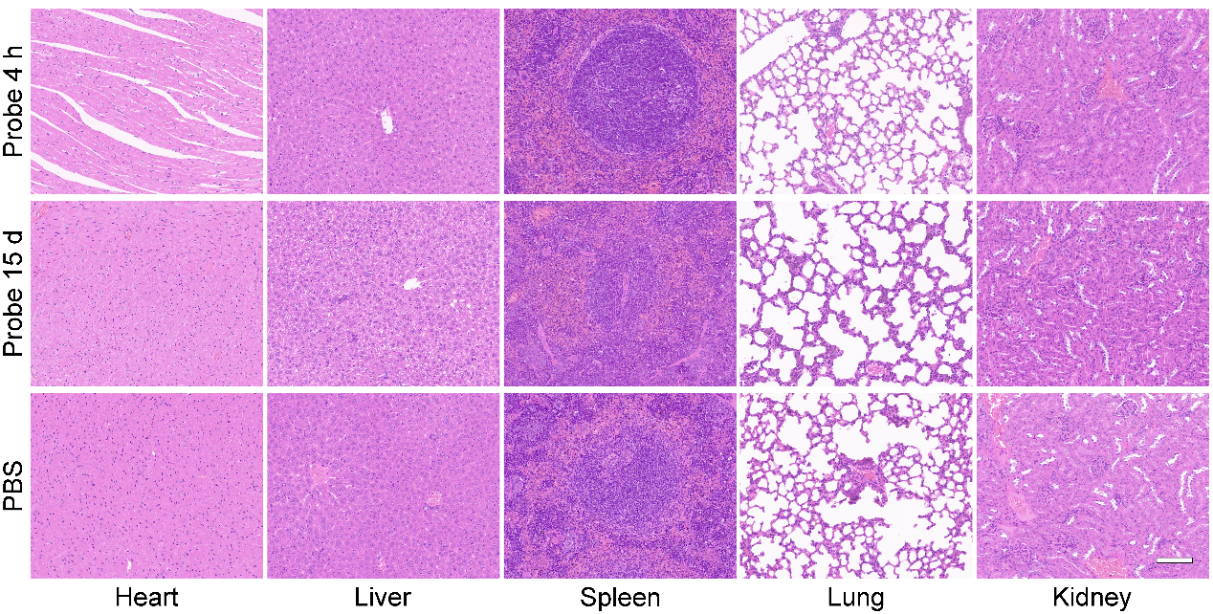


**Fig. S11** H&E staining of tissue slices from major organs of mice treated with Fe^III^TA@HA nanoprobes or PBS. The embedded scale bar corresponds to 100 μm.


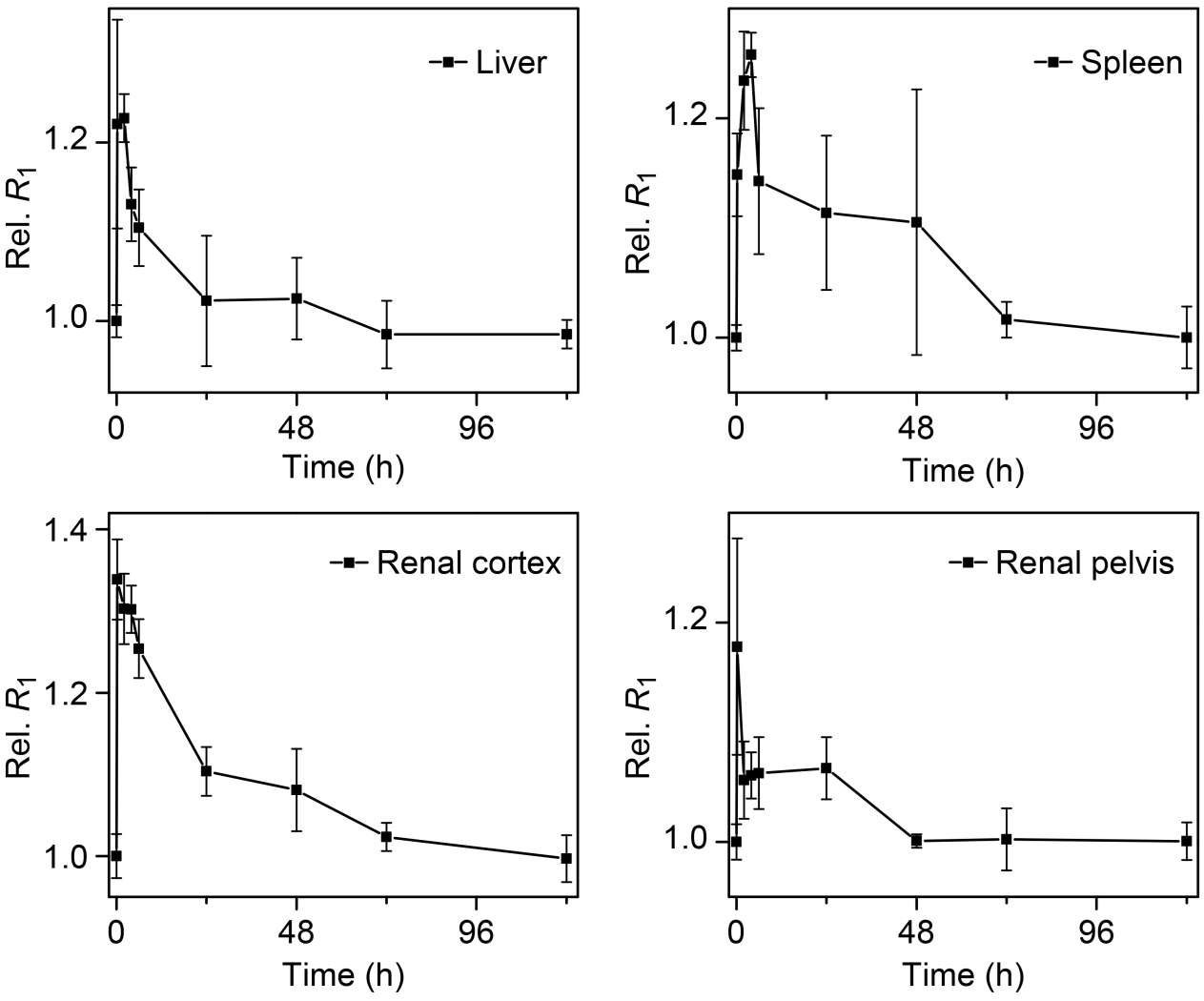


**Fig. S12** Temporal evolution of relative *R*_1_ values of the liver region, spleen region, renal cortex region, and renal pelvis region of mice. Data are plotted as the mean ± standard deviation, n = 3.

**Calculation of the photothermal conversion efficiency.**

Under the room temperature of 25 °C, 1.0 mM Fe^III^TA@HA nanoprobes solution (with respect to Fe^3+^) was loaded into a cuvette and irradiated using the 650 nm laser, followed by natural cooling after laser light was turned off. The temperature profile monitored were shown in Fig. 1f and 1g, respectively. The photothermal conversion efficiency is calculated according to the following equation.[^1^](#_ENREF_1) The photothermal conversion efficiency η can be given as

$$\eta=\frac{m\cdot c\cdot T_{max}-T_{max,water}}{I\cdot\left( 1-{10}^{-A} \right)\cdot\tau_{s}}$$

where m is the solution mass and equal to 0.1 g in the current study, c is the heat capacity of water and equal to 4.2 J/g, T_max_ and T_max,water_ are the maximum temperature change for nanoprobes solution and water, which are 44.9 °C and 0.9 °C, I is the laser power and equal to 1.0 W in the current study, A is the absorbance of nanoparticles solution at 650 nm and equal to 0.437, τ_s_ is the system time constant and equal to 197 s according to the linear regression of the cooling profile after laser irradiation (Fig. S4). The photothermal conversion efficiency of Fe^III^TA@HA nanoprobe is therefore calculated to be 14.8% at 650 nm laser irradiation by using these parameters.

**References**

1. X. Ding, C. H. Liow, M. Zhang, R. Huang, C. Li, H. Shen, M. Liu, Y. Zou, N. Gao, Z. Zhang, Y. Li, Q. Wang, S. Li and J. Jiang, *J. Am. Chem. Soc.*, 2014, **136**, 15684-15693.
